# Influence of angiotensin II type 1 receptors and angiotensin-converting enzyme I/D gene polymorphisms on the progression of Chagas’ heart disease in a Brazilian cohort: Impact of therapy on clinical outcomes

**DOI:** 10.1371/journal.pntd.0012703

**Published:** 2024-11-26

**Authors:** Thayse do Espírito Santo Protásio da Silva, Lucia Elena Alvarado-Arnez, Angelica Martins Batista, Silvia Marinho Martins Alves, Gloria Melo, Cristina Veloso Carrazzone, Isabelle de Oliveira Moraes, Antonio G. Pacheco, Camila Sarteschi, Milton Ozório Moraes, Wilson Oliveira Jr, Joseli Lannes-Vieira

**Affiliations:** 1 Laboratório de Biologia das Interações, Instituto Oswaldo Cruz/Fiocruz, Rio de Janeiro, Rio de Janeiro, Brazil; 2 Laboratório de Hanseníase, Instituto Oswaldo Cruz/Fiocruz, Rio de Janeiro, Rio de Janeiro, Brazil; 3 Ambulatório de Doença de Chagas e Insuficiência Cardíaca do Pronto Socorro Cardiológico de Pernambuco (PROCAPE)/UPE, Recife, Pernambuco, Brazil; 4 Instituto do Coração (InCor), Escola de Medicina, Universidade de São Paulo, São Paulo, São Paulo, Brazil; 5 Programa de Computação Científica, Fiocruz, Rio de Janeiro, Rio de Janeiro, Brazil; CSIR-Indian Institute of Chemical Biology, INDIA

## Abstract

Chagas disease (CD), a neglected tropical disease, is caused by infection by the protozoan *Trypanosoma cruzi*. One-third of CD patients develop cardiac disease (CARD), an inflammatory and fibrotic process that may progress to heart failure associated with reduced left ventricular ejection fraction (LVEF). The determinants of CD progression are still uncertain. In non-infectious conditions, the angiotensin-converting enzyme (ACE) functional insertion (I)/deletion (D) and type 1 angiotensin II receptor (AT_1_R) +1166A>C gene polymorphisms have been linked to clinical outcomes. In a Brazilian cohort of 402 patients with positive serology for CD, in a case-control study we used PCR for genotyping the *ACE* rs4646994 I/D and *AGTR1* rs5182C>T, rs275653 -119C>T, rs2131127A>G and rs5186 +1166A>C polymorphisms to evaluate association with CARD and progression to heart failure. Patients were classified as non-CARD (stage A; 109), and mild (stage B1; 161) or severe (stage C; 132) CARD. The groups were compared using unconditional logistic regression analysis and adjusted for non-genetic covariates (age, gender, and trypanocidal treatment). *ACE* II genotype appeared less frequent in C patients (15% in C *vs* 20% in B1 and 27% in A). After covariate adjustments, the *ACE* D allele showed a borderline association with susceptibility to severe CARD (C *vs* A: OR = 1.9; *P* = 0.08). *AGTR1* +1166AC genotype showed a borderline association with protection against the progression and severity of CARD (C *vs* A: OR = 0.6; *P* = 0.09; C *vs* B1: OR = 0.6; *P* = 0.07; C *vs* A + B1: OR = 0.6; *P* = 0.05). However, adjustments for multiple comparisons showed no association of *ACE* I/D and *AGTR1* polymorphisms with susceptibility and severity of CARD. The rs275653/rs2131127/rs5186/rs5182 T/A/C/T haplotype was protective against progression to the severe form of CARD (C *vs* B1: OR = 0.3; *P* = 0.03). Moreover, patients with *ACE* II and *AGTR1* rs5186 +1166AC genotypes presented higher LVEF%. In C patients, TNF serum levels were higher in *ACE* D carriers than in II genotype. Although limited in number, a cross-sectional observation suggests that C-stage patients treated with benznidazole years prior to administration of ACE inhibitors/AT_1_R antagonists show reduced TNF serum levels and improved LVEF%. Therefore, variants of *ACE* and *AGTR1* genes may influence the outcome of Chagas’ heart disease and should be explored in precision medicine. Further, pharmacotherapies may improve immunological abnormality and clinical outcome in CD patients. Altogether, these data support prospective studies of this cohort and replication in other cohorts.

## Author summary

It is estimated that 6–7 million individuals worldwide are infected with *Trypanosoma cruzi*, the etiologic agent of Chagas disease (CD), a neglected illness that impacts public health. Chronic cardiopathy is the most severe manifestation of CD, an inflammatory and fibrotic process that can progress to heart failure with reduced left ventricular ejection fraction (LVEF). Host genetics may be a determining factor in the progression of CD. In a group of chronic CD patients, we evaluated genetic polymorphisms of members of the renin-angiotensin system (*ACE* I/D; *AGTR1* variants), essential in regulating blood pressure and cardiovascular events. *ACE* D allele carriers and *AGTR1* rs5186 +1166AA genotype present worse LVEF. Thus, variants of ACE and AT_1_R genes may be involved in the outcome of Chagas’ heart disease. Although studying a low number of patients with severe cardiopathy, our data suggest that etiological treatment years before therapy with cardioprotectors (ACE inhibitors/AT_1_R blockers) reduced serum levels of TNF, an inflammatory cytokine important in cardiac pathology, and improved LVEF. This paves the way for *ACE* and *AGTR1* genotyping, and adoption of therapeutic regimens may be explored as strategies for precision medicine in CD patients. Replicating of these findings in other cohorts is essential.

## Introduction

Chagas disease (CD) is a neglected tropical disease caused by infection by the protozoan parasite *Trypanosoma cruzi*, typically transmitted in a vector-borne way. According to the World Health Organization, 6–7 million people are infected worldwide, mainly in endemic areas of Latin America [1]. In recent decades, the epidemiological profile of CD has changed due to immigration to North America, Europe, and Asia [2]. Prevention efforts focus on vector control, housing improvement and epidemiological surveillance, mainly based on screening of blood products [3], while controlling congenital and food-borne transmissions remain a challenge [4]. Regardless of promising candidates [5,6], there are currently no prophylactic or therapeutic vaccines to improve the prognosis of CD [7]. Benznidazole, the first-choice drug for etiological treatment, effectively reduces the parasite load in both acute and chronic phases of CD [8]. In the acute phase, cure confirmed by negative serology is achieved in 60–80% of cases [9,10]. However, as the acute infection is predominantly asymptomatic, valuable treatment time is often lost [9]. Moreover, we face the challenge of diagnosing and providing treatment and medical care to an estimated millions of chronically infected patients [4].

In the natural history of CD, the chronic phase’s clinical outcomes may range from the indeterminate form in 60–70% of the individuals to the cardiac form (CARD) varying from mild to severe in 20–30% of patients [10]. Moreover, progression from indeterminate to CARD may range from 0.2 to 10.3%, with an annual average of 1.9% [11]. The factors determining the different clinical outcomes in CD remain to be unveiled, especially for patients who will develop heart failure (HF) with reduced left ventricular ejection fraction (HFrEF), a severe form of CARD with a poor prognosis [12]. Like other chronic heart diseases, and based on the scenario of Chagas’ heart disease, studies on genetic biomarkers are mainly focused on genes that encode cytokines, chemokines, and critical molecules for effector immune response. However, controversial data have been obtained, and consensus on the association of genetic biomarkers with the risk of onset or progression of CARD is lacking [13–16].

Angiotensin I-converting enzyme (ACE) is a zinc-carboxydipeptidase in a vital regulator of blood pressure and vascular remodeling. ACE acts by converting the inactive angiotensin I to active angiotensin II, a potent vasoconstrictor member of the renin-angiotensin system (RAS) and an essential peptide for the pathophysiology of HF [17] and cardiac fibrosis [18]. ACE also metabolizes other molecules, and the potent vasodilator bradykinin is degraded by ACE activity [19]. *ACE* gene variants have been associated with poor echocardiographic outcomes in cardiomyopathies, risk of HF, and mortality, regardless of the etiology of the heart disease [20–22]. The most studied *ACE* polymorphism (rs4646994) corresponds to the insertion (I)/deletion (D) of 287 base pairs (bp) located in intron 16 that controls ACE expression throughout a mechanism that remains elusive [23,24]. *ACE* I/D polymorphism explains 47% of the variance in serum ACE activity [23]. Compared to *ACE* II, DD individuals have roughly twice the levels of ACE on the plasma membrane of leukocytes [25], and serum ACE concentration and activity [26]. DD genotype is also related to increased angiotensin II activity and bradykinin degradation [25]. More recent study sustained the tri-modal frequency distribution of ACE activity in serum expected to the *ACE* II, ID, DD genotypes, in the absence of ACE inhibitor (ACEi) [27]. Interestingly, *ACE* II genotype and lower plasma angiotensin II levels have been associated with a greater and longer lasting response to the hydrophilic ACEi enalapril [28]. Although the role of *ACE* I/D genotypes in clinical outcomes has been challenged [29], a recent systematic review supports to association of *ACE* I/D polymorphism with cardiovascular adversities [30]. In a Brazilian cohort, DD and DI genotypes were correlated with decreased left ventricular ejection fraction (LVEF) in patients with ischemic HF [31]. Alterations of RAS-controlled physiological processes are pivotal traits of Chagas’ heart disease [10]. A study with a limited number of Venezuelan CD patients revealed no association between *ACE* I/D polymorphism and CARD progression [32]. In a Brazilian cohort of 193 patients, *ACE* I/D variants did not help to establish a relation with clinical manifestations in HF secondary to CD [33]. More recently, we studied 343 Brazilian CD patients, showing that *ACE* DD genotype/D carriers were more prevalent in CARD patients with HF [16].

RAS’s main non-beneficial biological effects are mediated by sustained activation of the angiotensin II type 1 receptor (AT_1_R). Several single nucleotide polymorphisms (SNP) of the *AGTR1* gene have been described. Functionally, AT_1_R mRNA and protein expression is significantly higher for carriers of the *AGTR1* rs5186 +1166 CC genotype compared to AA and AC genotypes [34]. Furthermore, rs5186 +1166 AC/CC genotypes were more present in patients with left ventricular dysfunction than those without this alteration [35]. A recent study showed that the *AGTR1* rs275653 AA genotype reduced the risk of small artery occlusion strokes, although it did not affect AT_1_R expression [36]. Additionally, the *AGTR1* rs5182 CC genotype was correlated with a higher risk of developing hypertension in a cohort of Mexican individuals [37]. Functionally, AT_1_R stimulation induces endothelial dysfunction, increases vascular inflammation, and promotes atherosclerosis [38]. AT_1_R signaling also participates in arterial pressure disorders, heart inflammation, and fibrosis [39]. To date, *AGTR1* polymorphisms have not been evaluated in CD patients.

AT_1_R regulates the inflammatory response, leading to gene expression of cytokines, such as tumor necrosis factor (TNF) [40]. Serum TNF levels increased in non-infectious HF. Elevated serum levels of TNF were related to cachexia in patients with HF [41], while in ischemic cardiomyopathy, high TNF plasma concentrations were detected in patients with reduced LVEF [42]. In CD patients, increased TNF plasma concentrations were related to the severity of CARD and the degree of reduction of LVEF [43,44]. Moreover, in the chronic phase of infection, TNF is considered a fingerprint of the systemic inflammatory response and an essential biomarker of cardiac disorders in individuals with chronic chagasic cardiomyopathy [12,45]. Nonetheless, more recent studies challenged this concept [14,46].

In the present study, a group of 402 CD patients from Northeast Brazil was classified according to the I Latin American Guideline for Chagas disease [47] and evaluated for association of *ACE* I/D and *AGTR1* rs 5182C>T, rs 275653C>T, rs 2131127A>G and rs 5186+1166A>C as risk factors for CARD, and progression to the severe form, particularly with HFrEF. In CD, ACEi and angiotensin receptor blockers (ARB) are recommended to treat chronically infected patients according to indicative clinical abnormalities [4,10,47]. In a cross-sectional observational study, we assessed the effects of the etiological treatment with benznidazole and therapies with ACEi/ARB on the TNF serum levels and the degree of LVEF dysfunction.

## Methods

### Ethical statement

This study was carried out in accordance with the recommendations of the Ethics Committees of Fiocruz/RJ (541/09) and PROCAPE/UPE (80210/10). All participants provided written informed consent in accordance with the Declaration of Helsinki.

### Study population

A group of 402 patients residing in the Northeast of Brazil was admitted in a case-control association study during a period of 5 years (2010–2015) at the Chagas Disease and Heart Failure Outpatient Clinic of the Cardiology Emergency Department of Pernambuco (PROCAPE)/University of the State of Pernambuco (UPE). At enrollment, 10 mL of peripheral blood was collected to confirm serological diagnosis and DNA isolation. According to the “Brazilian Consensus on Chagas disease” (2005) of the Brazilian Health Ministry [48], the serological diagnosis of CD was determined by at least two independent tests, including enzyme-linked immunosorbent assay (ELISA) and indirect immunofluorescence, performed by the Central Reference Laboratory (LACEN) from Pernambuco, Brazil. The study did not include patients under 18 years of age diagnosed with digestive and cardiodigestive forms of CD or diagnosed with co-infections, alcohol users, and vulnerable populations (pregnant, Indigenous, Quilombola, and chronic mental illness patients). In this cross-sectional observational study, patients who consented to participate were evaluated by anamnesis, and the 12-lead electrocardiogram (ECG; Ecafix, São Paulo, SP, Brazil) findings were recorded. Echocardiography (ECHO), two-dimensional Doppler, and M-mode imaging were performed using a Vivid 3 (GE Health Care, Wauwatosa, WI, USA) with digitally recorded images. Participants were classified according to the “I Latin American Guideline for the Diagnosis and Treatment of Chagas Heart Disease” [47]. As previously described [14–16], and according to the evaluated criteria, patients were classified as follows: stage A, a group of 109 patients without cardiac symptoms and with normal ECG and ECHO recordings; stage B1, a group of 161 patients without clinical signs of HF, ECG or ECHO with segmental dysfunction, but with normal ventricular function; and stage C, a group of 132 patients with clinical signs of HF, electrocardiographic changes and structural cardiomyopathy by ECHO assessment, including reduced LVEF (<45%; Simpson’s method). Etiological treatment with the trypanocidal drug benznidazole, recommended to chronically infected patients [48] was administered from 1 to 20 years before the inclusion of the patients in the present study. Prescription and doses of other medications (ACEi, ARB, beta-blocker, diuretic, others) were recommended according to individual clinical abnormalities [47]. All data were obtained from Patient Registries. The **S1 Table** shows individual epidemiological and clinical classifications and raw genotyping data of the 402 evaluated CD patients. The **S2 Table** shows all data used to generate graphs.

### DNA processing and genotyping

Genomic DNA was extracted from frozen blood samples using a modified salting-out precipitation technique [49]. DNA from each sample was quantified using NanoDrop ND-1000 Spectrophotometer (NanoDrop Technologies, USA). The *ACE* I/D polymorphism (rs4646994) was evaluated by conventional PCR using 25 ng yield DNA for each sample and 0.1 μM of the 5’CTGGAGACCACTCCCATCCTTTCT3’ and 5′GATGTGGCCATCACATTCCGTCAGAT3′ primers with KAPA2G Fast HotStart PCRMix (Merck, USA) for a final reaction of 25 μL. The PCR conditions were as follows: 95°C/3 minutes; 35 cycles of 95°C/15s, 66°C/15s and 72°C/5s, adapted from a previous publication [50]. The products were submitted to 2% agarose gel electrophoresis. The genotyping was based on product sizes: a 490 bp fragment for genotype II; two fragments, one of 490 and other of 190 bp for the heterozygous DI genotype; and a 190 bp fragment for the DD genotype. *AGTR1* SNPs were analyzed using TaqMan genotyping assays: +573 C>T rs5182, -119 C>T rs275653, rs2131127, +1166 A>C rs5186 (**S3 Table**). Reactions were performed with 30 ng yield DNA for each sample following the manufacturer’s recommendations for allelic discrimination on the ViiA 7 Real-Time PCR System (Applied Biosystems, USA).

### Serum levels of TNF

According to the manufacturer’s instructions, the serum levels of TNF were quantified using ELISA DuoSet (R&D Biosystems, Minneapolis, MN, USA). Data were analyzed by comparing the levels of TNF and (i) the clinical groups, (ii) the *ACE* genotypes, (iii) benznidazole-treatment administered to clinical groups (A, B1, C), and (iv) cardioprotective therapeutic schemes administered to benznidazole-treated C group of patients.

### Statistical analysis

The dependent variable was the stage of Chagas’ heart disease (stages A, B1, and C), according to the I Latin American Guidelines for the diagnosis and treatment of Chagas cardiomyopathy [47]. The genotype, carriers of alleles, or minor alleles were determined by direct counting. The chi-square test determined Hardy-Weinberg equilibrium. The risk of progression of Chagas’ heart disease was evaluated by comparing individuals at different stages of the cardiac form of CD disease, including non-genetic covariates such as age, and gender. The logistic regression model was used to analyze previous trypanocidal treatments. Association tests are presented as odds ratios (OR) with the respective 95% confidence interval (CI). Adjustment for multiple comparisons was performed using the false discovery rate (FDR). The analyses were performed in R version 3.3.3 environment using the following packages: coin, epiDisplay, gap, genetics, stats, haplo.stats, meta, metafor and SNPassoc. Comparisons between demographic or clinical variables were performed using the chi-square or Kruskal-Wallis test when appropriate, and the frequencies of each variable excluded missing data. First, we tried to determine the influence of *ACE* variants and *ATGR1* SNPs on the development of the cardiac form of CD. Patients in group A were considered as controls, while patients in groups B1 and C were considered as cases. So, to examine the influence of gene variants and SNPs on the severity of CD cardiomyopathy, group B1 (mild cardiopathy) was considered a control. In contrast, those in group C (severe cardiopathy) were considered as cases. For polymorphisms located within the same gene, haplotype frequencies were estimated using the maximum likelihood method and compared using the same unconditional regression models used to analyze individual SNPs. A linkage disequilibrium (LD) analysis was assessed using the r2 statistic using Haploview software. LVEF% and genotypes, LVEF% and treatments, TNF serum levels, and TNF serum levels and treatments were analyzed using GraphPad Instat software version 8.0 (San Diego, CA). The Shapiro-Wilk test was used to assess the normality of the data. Differences were analyzed using the Student *t*-test for normally distributed data formed by two groups. For normally distributed data of more than two groups, the difference between groups was analyzed using the parametric one-way ANOVA test, corrected with Turkey post hoc test with multiple comparisons, with a 95% confidence. *P* values ≤ 0.05 were considered statistically significant.

## Results

### Epidemiological characteristics of the study groups

In the studied cohort, all admitted patients had two positive serology tests for CD. **Table 1** presents the main characteristics of the studied patients. For simplicity, we annotate the sequential results showing comparisons between case-control groups as B1 (case) versus A (control), C (case) versus B1 (control), and C (case) versus A (control). Comparisons of age (A:51 ± 12 years; B1: 60 ± 12 years; and C: 60 ± 11 years) showed that groups C and B1 are similar (*p* > 0.05), while B1 *vs* A (*p* < 0.001) and C *vs* A (*p* < 0.001) show differences. In all groups, most patients are female. Considering the frequencies of gender, B1 *vs* A and C *vs* A are comparable (*p* > 0.05), while C *vs* B1 are different groups (*p* < 0.01). Comparison of other characteristics such as ethnicity (B1 *vs* A, *p* < 0.01; C *vs* B1, *p* < 0.01; and C *vs* A, *p* > 0.05), education levels (B1 *vs* A, *p* < 0.001; C *vs* B1, *p* < 0.001; and C *vs* A, *p* > 0.05) and minimum wage income (B1 *vs* A, *p* < 0.001; C *vs* B1, *p* < 0.001; and C *vs* A, *p* > 0.05) also revealed differences between the groups. Most individuals were born and resided in different geographic areas of the State of Pernambuco (PE, 79.2%), with a higher frequency of patients in groups B1 (51%; *p* < 0.001) and C (43%; *p* < 0.001) born in the Mata area. Patients born in other states in the Northeast region and other Brazilian States but residing in the State of Pernambuco (20.8%) were also admitted in our study.

**Table 1:** Demographic and epidemiological data of the patients recruited for the present study.

Variable^a^	A Group N = 109	B1 Group N = 161	C Group N = 132	P-value
**Gender**				
Female	71 (65.1%)	123 (76.4%)	79 (59.8%)	Chisq. 0.008
Male	38 (34.9%)	38 (23.6%)	53 (40.2%)	
**Age** (years)^b^ **(n = 400)**	51 ± 12	60 ± 12	60 ± 11	KW < 0.001
≤ 45	37 (33.9%)	23 (14.5%)	16 (12.1%)	
>45	72 (66.1%)	136 (85.5%)	116 (87.9%)	
**Ethnicity (n = 353**)				Chisq. 0.32
Black	9 (9.6%)	13 (9.2%)	13 (11.1%)	
Mestizo	58 (61.7%)	103 (72.5%)	77 (65.8%)	
White	27 (28.7%)	26 (18.3%)	27 (23.1%)	
**Monthly income (n = 347)**				Chisq. 0.006
Up to 1 MW^c^	68 (68.7%)	106 (79.1%)	94 (82.5%)	
2–4	14 (14.1%)	22 (16.4%)	13 (11.4%)	
More than 5	17 (17.2%)	6 (4.5%)	7 (6.1%)	
**Education (n = 375)**				Chisq. 0.14
Up to 4 years	83 (81.4%)	120 (78.9%)	107 (88.4%)	
More than 4 years	19 (18.6%)	32 (21.1%)	14 (11.6%)	
**Region of birth (n = 380)**				Chisq. < 0.001
PE, Agreste	26 (24.5%)	22 (14.4%)	31 (25.6%)	
PE, Mata	22 (20.8%)	78 (51%)	52 (43%)	
PE, Metropolitan	6 (5.7%)	8 (5.2%)	6 (5.0%)	
PE, Sertão	30 (28.3%)	31 (20.3%)	14 (11.6%)	
Other States^d^	22 (20.8%)	14 (9.2%)	18 (14.9%)	

^a^ Results are presented as number (frequency)

^b^ Mean and ± standard deviation

^c^ MW, minimum wage: U$ 250-350/month in the period of 2010–2015

^d^ Other states of birth include: Northeast (Alagoas, Bahia, Paraiba, Piauí, Rio Grande do Norte) and other regions (Matogrosso, Minas Gerais and São Paulo)

Abbreviations: N, Total count of patients with available information for the referred variable; PE, State of Pernambuco; KW, Kruskal-Wallis test

### Clinical information and medication use

**Fig 1:**
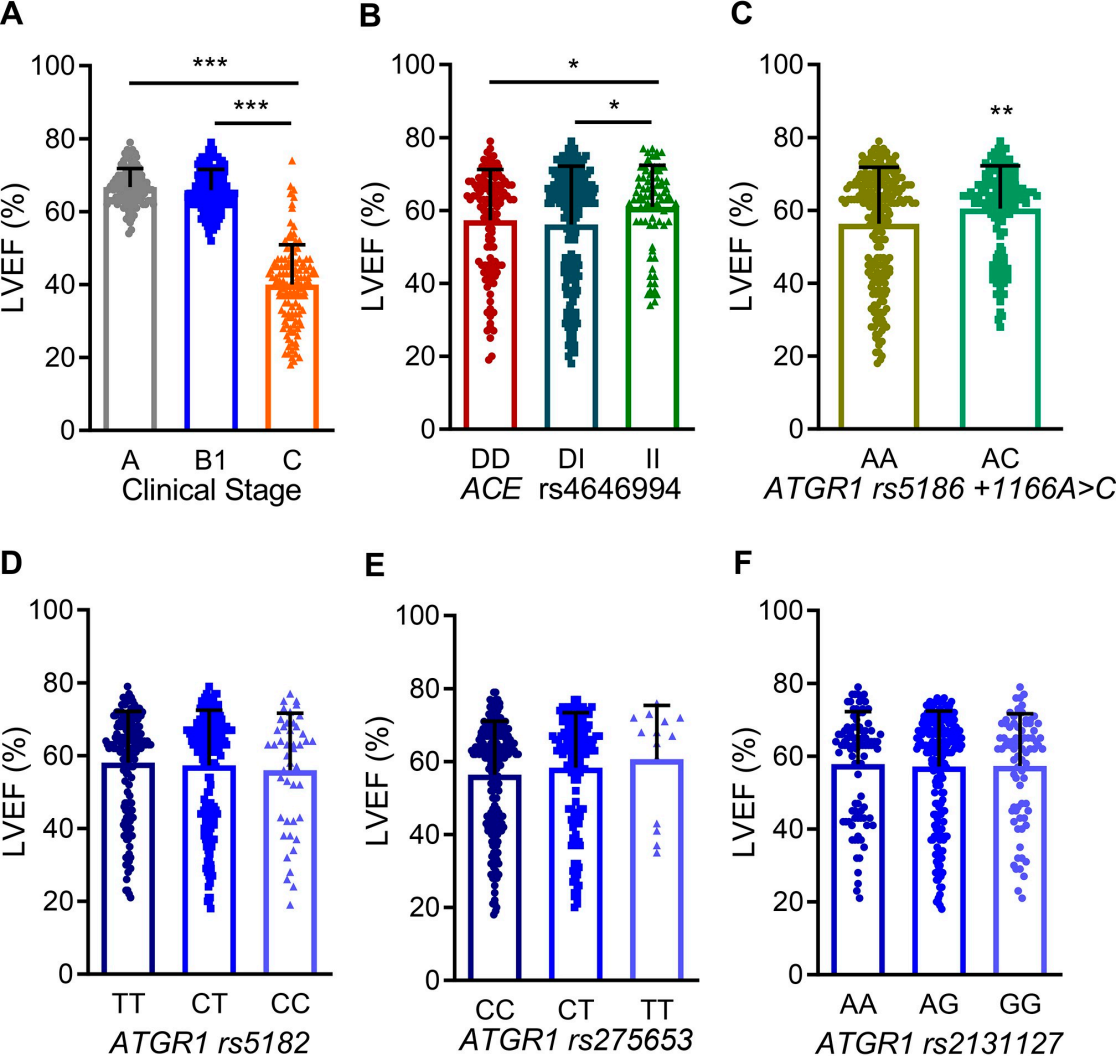
Association between ACE and AT_1_R genetic polymorphisms and left ventricular ejection fraction. All CD patients were grouped according to clinical classification (non-cardiopathic–stage A, 109 patients; mild–stage B1, 161 patients; and severe–stage C, 132 forms of CARD) and genotyped for *ACE* rs4646994 insertion (I)/deletion (D) and *ATGR1* SNPs rs5182C>T, rs275653 -119C>T and rs2131127A>G polymorphisms. **A-F**. Simpson’s left ventricular ejection fraction (LVEF%) is shown according to the *ACE* and *ATGR1* genotypes. The two-groups difference was analyzed with the Student *t*-test. For data from more than two-groups, the difference between groups was analyzed using the parametric one-way ANOVA test, corrected with Turkey post hoc test with multiple comparisons. Each dot represents a patient. Data show means ± SD. *, *p* < 0.05; **, *p* < 0.01, ***, *p* < 0.001.

**Table 2** and **Fig 1A** show that LVEF was lower in stage C patients (40 ± 10.9%) compared to B1 (65.7 ± 6.5%) and A (66.7 ± 5%) patients (C *vs* B1, *p* < 0.001; and C *vs* A, *p* < 0.001; B1 *vs* A, *p* > 0.05), as intrinsic characteristics of their clinical stages. According to indicative clinical abnormalities, the use of drugs such as ACEi (mainly captopril, enalapril) or ARB (mainly losartan) was prescribed to 56.1% of patients in group C (showing HFrEF), 23.6% of patients of group B1 and 13.8% of group A (C *vs* B1, *p* < 0.001; and C *vs* A, *p* < 0.001; B1 *vs* A, *p* < 0.05). Beta-blockers (mainly atenolol and propranolol) were prescribed to 53% of patients in group C, 12.4% of patients of group B1 and 9.2% of group A (C *vs* B1, *p* < 0.001; and C *vs* A, *p* < 0.001; B1 *vs* A, *p* > 0.05). In addition, the diuretic spironolactone was prescribed to 37.1% of patients in group C. It is important to note that the use of the trypanocidal drug benznidazole was recorded in 12.1% of stage C patients, 10.6% of stage B1, and 32.1% of stage A (C *vs* B1, *p* > 0.05 and C *vs* A, *p* < 0.001; B1 *vs* A, *p* < 0.001).

**Table 2:** Clinical information and the use of medications by the patients in each clinical form included in the study.

Variable^a^	A Group N = 109	B1 Group N = 161	C Group N = 132	
LVEF (%)^b^	66.7 ± 5.0	65.7 ± 6.5	40 ± 10.9	KW < 0.001
ACE inhibitors/ARB	15 (13.8%)	38 (23.6%)	74 (56.1%)	
Beta-blocker	10 (9.2%)	20 (12.4%)	70 (53%)	
Spironolactone	1 (0.9%)	0 (0%)	49 (37.1%)	
Benznidazole	35 (32.1%)	17 (10.6%)	16 (12.1%)	

^a^ Results are presented as number (frequency)

^b^ Mean and ± standard deviation

Abbreviation: LVEF, left ventricular ejection fraction

### Association between ACE and AT_1_R gene polymorphisms and heart failure with reduced left ventricular ejection fraction

Our research has found a significant relation between severe CARD (stage C) and lower LVEF% (**Table 2** and **Fig 1A)**. Our primary objective was to unravel the role of the *ACE* and *ATGR1* genotypes in this trait, irrespective of the clinical stage of Chagas’ heart disease. The discovery that carriers of the *ACE* rs4646994 II genotype exhibit higher LVEF% compared to *ACE* DD (*p* < 0.05) and *ACE* DI (*p* < 0.05) genotypes (**Fig 1B**) is a crucial step in addressing the complex question of HF in CD. Following the same reasoning, the analysis of *ATGR1* SNP variants showed that rs5186 +1166A>C heterozygous patients had higher LVEF% compared to AA patients (**Fig 1C**). The investigation of the other *ATGR1* SNP variants (rs5182 C>T, rs275653–119 C>T, rs2131127 A>G) showed no relation with LVEF% in this cohort of CD patients (**Fig 1D–1F**).

### Polymorphisms at ACE and AT_1_R genes and association with Chagas’ heart disease

Genotypic frequencies were distributed according to the Hardy-Weinberg equilibrium (*p* > 0.05) for all tested groups. All genotyped polymorphisms had a call rate efficiency of at least 95%. Unconditional logistic regression results comparing patients in stage B1 (case) with those in stage A (control) showed no significant differences in any genotype, allelic, or carrier comparison for the *ACE* rs4646994 I/D polymorphism. Subsequently, when comparing stage C patients (case) with stage A patients (control), we found that the *ACE* DI or DD genotypes and D carriers were associated with the risk of developing HF (*P* = 0.02). However, after statistical adjustments for gender, age, and use of the trypanocidal drug benznidazole, *ACE* DI genotype (*P* = 0.07) and D carriers (*P* = 0.08) showed a borderline association with susceptibility to severe CARD (**Table 3**), such indication of a potential association was lost after adjustments for multiple comparisons (**Table 3**).

**Table 3:** Analysis of *ACE* rs4646994 I/D polymorphism.

				A versus B1		A versus C		A versus B1 + C		B1 versus C		A + B1 versus C	
	Stage A N = 109	Stage B1 N = 154	Stage C N = 129	OR^a^ [95% CI]	*P*-value^b^	OR^a^ [95% CI]	*P*-value^b^	OR^a^ [95% CI]	*P*-value^b^	OR^a^ [95% CI]	*P*-value^b^	OR^a^ [95% CI]	*P*-value^b^
*ACE* rs4646994 *I/D*													
II	29 (0.27)	31 (0.20)	19 (0.15)						- Reference -				
ID	45 (0.41)	73 (0.47)	63 (0.49)	1.3 [0.6–2.5]	0.40 (0.65)	1.8 [0.9–3.8]	**0.07** (0.22)	1.5 [0.8–2.8]	0.12 (0.32)	1.3 [0.7–2.6]	0.52 (0.65)	1.5 [0.8–2.8]	0.17 (0.40)
DD	35 (0.32)	50 (0.33)	47 (0.36)	1.2 [0.6–2.5]		1.9 [0.9–4.1]		1.5 [0.8–2.8]		1.5 [0.7–3.0]		1.6 [0.9–3.1]	
D carriers	80 (0.73)	123 (0.80)	110 (0.85)	1.2 [0.7–2.3]	0.49 (0.80)	1.9 [0.9–3.7]	**0.08** (0.21)	1.5 [0.9–2.6]	0.16 (0.32)	1.4 [0.7–2.6]	0.35 (0.47)	1.6 [0.9–2.8]	0.12 (0.21)

^a^ Odds ratio (OR) values shown are corrected for gender, age and trypanocidal treatment.

^b^ Values in parentheses correspond to *P-values* after multiple comparisons (FDR) adjustments.

Results are shown as n (frequency).

Abbreviations: CI = confidence interval.

Total genotype counts can vary due to different genotypic call rates.

Considering the biological relevance of AT_1_R for the deterioration of cardiac function in several clinical conditions [39], we decided to evaluate the association of *AGTR1* SNP polymorphisms with the susceptibility to CARD and the severe CARD with HF. Analysis of *AGTR1* SNPs rs5182C>T, rs275653 -119C>T, and rs2131127 A>G did not show significant associations with susceptibility (B1 *vs* A; C *vs* A; B1 + C *vs* A) or severity of (C *vs* B1; C *vs* A + B1) to CARD in our group of CD patients (**Table 4**). The *AGTR1* rs5186 +1166 AC heterozygotes showed a borderline association with protection against Chagas’ heart disease (C *vs* A: OR = 0.6; *P* = 0.09; C *vs* B1: OR = 0.6; *P* = 0.07; C *vs* A + B1: OR = 0.6; *P* = 0.05), as shown in **Table 4**. Again, such putative associations were lost after adjustments for multiple comparisons (**Table 4**). Considering ATGR SNPs as a block (**S1 Fig**), it is essential to highlight that the logistic regression analysis (**Table 5**) showed that the *AGTR1* rs275653/rs2131127/rs5186/rs5182 T/A/C/T haplotype was protective against progression to the severe CARD (C *vs* B1: OR = 0.3; *P* = 0.03).

**Table 4:** Analysis of single nucleotide polymorphisms located in *AGTR1* gene.

				A versus B1		A versus C		A versus B1 + C		B1 versus C		A + B1 versus C	
	Stage A N = 110	Stage B1 N = 163	Stage C N = 133	OR^a^ [95% CI]	*P*-value^b^	OR^a^ [95% CI]	*P*-value^b^	OR^a^ [95% CI]	*P*-value^b^	OR^a^ [95% CI]	*P*-value^b^	OR^a^ [95% CI]	*P*-value^b^
***ATGR1* +573 C>T rs5182**													
TT	44 (0.41)	73 (0.46)	47 (0.37)					- Reference -					
CT	50 (0.47)	70 (0.44)	62 (0.49)	0.9 [0.5–1.6]	0.65 (0.65)	1.2 [0.6–2.2]	0.85 (0.85)	1.1 [0.7–1.9]	0.77 (0.77)	1.3 [0.8–2.2]	0.24 (0.40)	1.3 [0.8–2.1]	0.32 (0.40)
CC	13 (0.12)	15 (0.10)	18 (0.14)	0.7 [0.3–1.6]		1.1 [0.5–2.7]		0.9 [0.4–1.9]		1.9 [0.8–4.2]		1.6 [0.8–3.2]	
C carriers	63 (0.60)	85 (0.54)	80 (0.63)	0.9 [0.5–1.5]	0.60 (0.80)	1.2 [0.7–2.1]	0.58 (0.58)	1.1 [0.7–1.8]	0.76 (0.76)	1.4 [0.9–2.4]	0.15 (0.30)	1.4 [0.9–2.1]	0.16 (0.21)
***ATGR1*–119 C>T rs275653**													
CC	56 (0.54)	85 (0.55)	80 (0.63)					- Reference -					
CT	46 (0.44)	60 (0.39)	43 (0.34)	0.9 [0.5–1.6]	0.17 (0.42)	0.6 [0.4–1.1]	0.34 (0.42)	0.7 [0.4–1.2]	0.18 (0.32)	0.7 [0.4–1.2]	0.20 (0.40)	0.7 [0.5–1.1]	0.25 (0.40)
TT	2 (0.02)	10 (0.06)	4 (0.03)	3.9 [0.7–20.3]		1.0 [0.1–6.4]		2.3 [0.4–11.8]		0.4 [0.1–1.5]		0.6 [0.2–1.8]	
T carriers	48 (0.46)	70 (0.45)	47 (0.37)	1.0 [0.6–1.8]	0.92 (0.92)	0.7 [0.4–1.2]	0.16 (0.21)	0.8 [0.5–1.3]	0.30 (0.40)	0.7 [0.4–1.1]	0.11 (0.30)	0.7 [0.4–1.1]	0.10 (0.21)
***ATGR1* rs2131127**													
AA	34 (0.32)	36 (0.23)	34 (0.27)					- Reference -					
GA	48 (0.45)	86 (0.56)	66 (0.52)	1.8 [1.0–3.4]	0.16 (0.42)	1.7 [0.9–3.2]	0.30 (0.42)	1.7 [1.0–3.0]	0.19 (0.32)	0.8 [0.5–1.5]	0.79 (0.79)	1.0 [0.6–1.8]	0.95 (0.95)
GG	25 (0.23)	32 (0.21)	27 (0.21)	1.4 [0.6–2.9]		1.4 [0.6–3.0]		1.4 [0.7–2.8]		0.8 [0.4–1.6]		1.0 [0.5–1.8]	
G carriers	73 (0.68)	118 (0.77)	93 (0.73)	1.7 [0.9–3.0]	0.08 (0.32)	1.6 [0.9–2.9]	0.14 (0.21)	1.6 [0.9–2.7]	**0.08** (0.32)	0.8 [0.5–1.4]	0.50 (0.50)	1.0 [0.6–1.7]	0.93 (0.93)
*ATGR1* +1166 **A>C rs5186**													
AA	60 (0.57)	86 (0.56)	87 (0.68)					- Reference -					
AC	46 (0.43)	67 (0.44)	40 (0.32)	1.2 [0.7–2.0]	0.54 (0.65)	0.6 [0.3–1.1]	**0.09** (0.22)	0.9 [0.5–1.4]	0.53 (0.66)	0.6 [0.4–1.0]	**0.07** (0.35)	0.6 [0.4–1.0]	**0.05** (0.25)

^a^ Odds ratio (OR) values shown are corrected for gender, age and benznidazol treatment.

^b^ Values in parentheses correspond to *P-values* after multiple comparisons (FDR) adjustments.

Results are shown as n (frequency).

Abbreviations: CI = confidence interval.

Total genotype counts can vary due to different genotypic call rates.

**Table 5:** Logistic regression results for haplotype analysis of SNPs that constitute the *ATGR1* cluster. **Haplotype frequencies are shown by group**.

rs275653/rs2131127/rs5186/rs5182				A versus B1		A versus C		A versus B1 + C		B1 versus C		A+ B1 versus C	
	Stage A N = 109	Stage B1 N = 161	Stage C N = 132	OR^a^ [95% CI]	*P*-value	OR^a^ [95% CI]	*P*-value	OR^a^ [95% CI]	*P*-value	OR^a^ [95% CI]	*P*-value	OR^a^ [95% CI]	*P*-value
C/A/A/T	0.09	0.07	0.09				- Reference -						
C/A/A/C	0.13	0.17	0.19	1.3 [0.5–3.6]	0.64	1.7 [0.6–5.0]	0.37	2.5 [0.8–8.0]	0.12	0.8 [0.3–2.1]	0.73	1.0 [0.5–2.3]	0.90
C/A/C/T	0.16	0.07	0.09	0.5 [0.1–2.0]	0.32	0.5 [0.2–1.7]	0.27	0.7 [0.2–2.3]	0.53	1.1 [0.3–4.3]	0.84	0.7 [0.2–2.0]	0.49
C/G/A/C	0.16	0.09	0.16	0.6 [0.2–1.6]	0.29	1.0 [0.4–2.8]	0.93	1.0 [0.4–2.8]	0.98	1.3 [0.5–3.6]	0.58	1.1 [0.5–2.4]	0.86
C/G/A/T	0.18	0.27	0.24	1.4 [0.5–4.1]	0.47	1.4 [0.5–4.0]	0.49	2.0 [0.7–5.6]	0.20	0.6 [0.2–1.6]	0.36	0.8 [0.4–1.8]	0.60
C/G/C/T	0.04	0.06	0.03	1.9 [0.5–7.5]	0.35	0.7 [0.2–3.1]	0.63	1.1 [0.3–4.1]	0.90	0.3 [0.1–1.1]	0.06	0.4 [0.1–1.5]	0.18
T/A/A/C	0.06	0.05	0.04	1.0 [0.2–4.1]	0.96	0.3 [0.06–1.4]	0.13	0.7 [0.2–2.8]	0.59	0.7 [0.2–3.0]	0.67	0.5 [0.2–1.8]	0.32
T/A/A/T	0.09	0.07	0.08	0.5 [0.1–2.0]	0.31	0.94 [0.2–4.3]	0.94	1.2 [0.2–7.0]	0.81	0.7 [0.2–2.7]	0.61	0.8 [0.3–2.3]	0.64
**T**/A/**C**/T	0.02	0.08	0.04	5.7 [0.7–4.9]	0.12	1.5 [0.2–9.4]	0.64	3.0 [0.5–17.0]	0.20	**0.3 [0.07–0.9]**	**0.03**	0.5 [0.2–1.4]	0.19
T/G/A/T	0.07	0.06	0.05	1.2 [0.4–4.5]	0.76	1.1 [0.3–4.4]	0.90	1.8 [0.5–7.1]	0.38	0.8 [0.2–2.5]	0.66	0.8 [0.3–2.2]	0.66

^a^ Odds ratio (OR) values shown are corrected for gender, age, and trypanocidal treatment.

Abbreviations: CI = confidence interval

### TNF levels related with *ACE* I/D and *ATGR1* rs5186 +1166A>C polymorphisms

**Fig 2:**
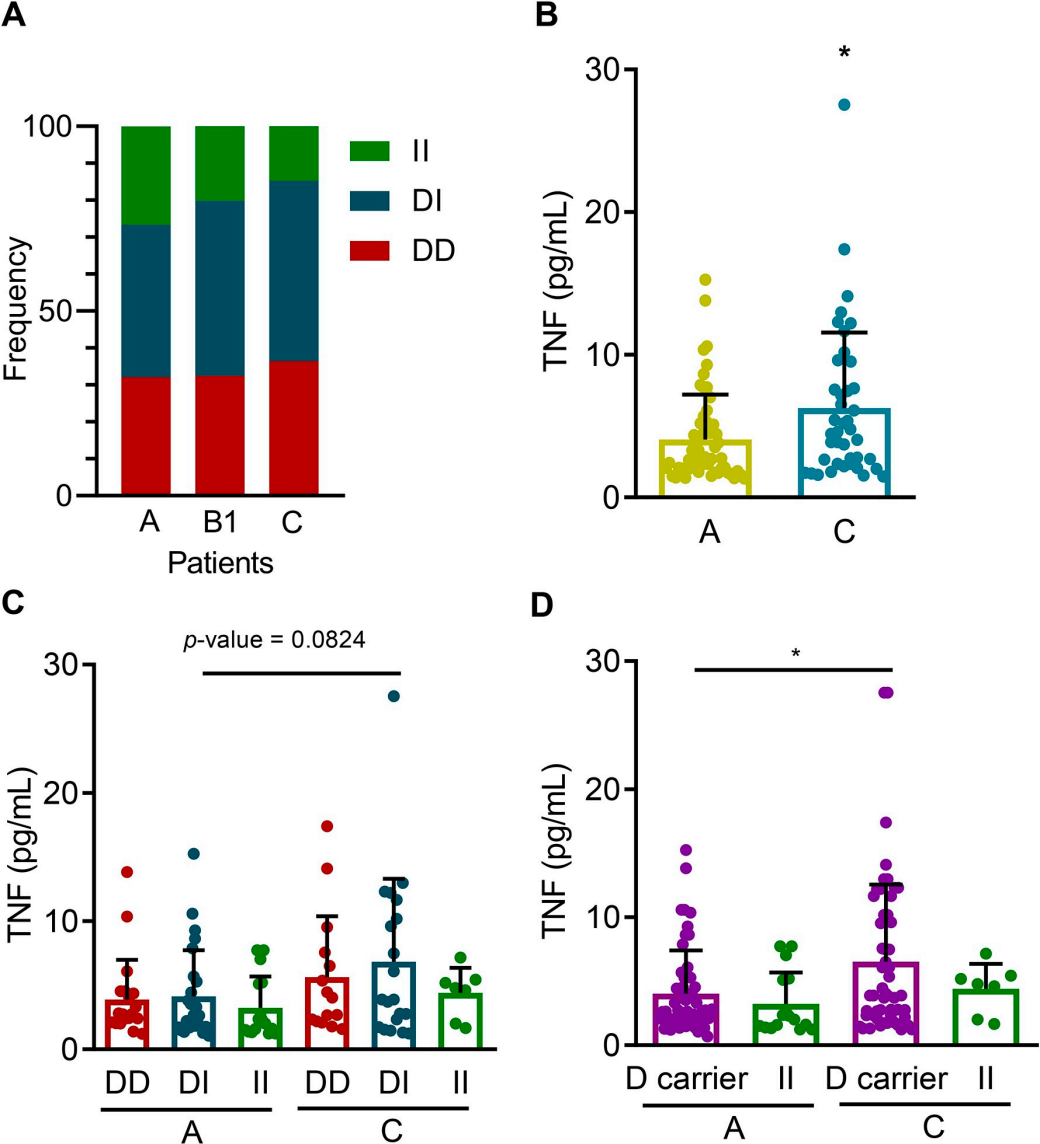
Genotype frequencies and TNF serum levels related to *ACE* I/D polymorphism in Chagas disease patients. All CD patients were grouped according to clinical classification (non-cardiopathic–stage A; CARD: mild–stage B1 patients; and severe–stage C) and genotyped for *ACE* rs4646994 insertion (I)/deletion (D) polymorphism. TNF serum concentrations (pg/mL) are shown according to clinical classification, and the described *ACE* I/D genotypes. **A**. Frequencies of *ACE* DD, DI, and II genotypes in A, B1 and C patient groups. **B.** TNF serum levels in A (n = 56) and C (n = 41) patient groups. **C.** TNF serum levels in A (n = 20, 25, 16) and C (n = 15, 10, 7) patient groups bearing the *ACE* DD, DI, and II genotypes. **D.** TNF serum levels in A and C patient groups, classified as *ACE* D allele carriers and II genotypes. The difference between the two groups was analyzed using the Student *t*-test. For data from more than two groups, the difference between groups was analyzed using the parametric one-way ANOVA test, corrected with Turkey post hoc test with multiple comparisons. Each dot represents a patient. Data show means ± SD. *, *p* < 0.05.

The frequencies of DD, DI and II genotypes were dissimilar among the stage A (32% DD, 41% DI, 27% II), B1 (33% DD, 47% DI, 20% II) and C (36% DD, 49% DI, 15% II) CD patients (**Table 3**), with an apparent reduction of the frequency of II genotype in patients with severe CARD in the C group, compared with the non-CARD A group (**Fig 2A**). Thus, considering the contribution of RAS to inflammation [38] and the association of systemic inflammatory profile with the severity of CARD [46,51], we explored the relation of *ACE* genotypes and TNF serum levels in our group of patients. TNF levels were increased in C patients, compared to A patients (**Fig 2B**), and tended towards an increase in C patients with the DD and DI genotypes (**Fig 2C**). Moreover, TNF levels were elevated in D carriers but not in patients with the II genotype in C patients, compared to their counterparts in the group of A patients (**Fig 2D**). Also, in B1 patients TNF levels tended to be higher in DD and DI genotypes and were increased in D carriers compared to II genotype (**S2A and S2B Fig**). Regarding the *ATGR1* rs5186 +1166A>C genotypes, C group patients bearing AA and AC genotypes showed similar TNF serum levels (**S2C Fig**).

### Effect of treatment with benznidazole on TNF serum levels and LVEF

Initially, in a cross-sectional observational study we registered the impact of the treatment with the trypanocidal drug benznidazole on LVEF% and TNF serum levels. Benznidazole treatment was prescribed 1 to 20 years before the patient’s inclusion. As shown in **S3A Fig**, we found no significant difference in LVEF% when patients classified as non-cardiopathic (A) and cardiopathic with HFrEF (C patients) treated with benznidazole were compared to their respective counterparts not-treated patients. The finding of elevated TNF serum levels in not-treated C patients compared to not-treated A patients, and the comparable TNF levels in benznidazole-treated C patients to those found in not-treated and benznidazole-treated A patients (**S3B Fig**) may underscore the potential of benznidazole in improving outcomes.

### Impact of cardioprotective therapies in benznidazole-treated patients

**Fig 3:**
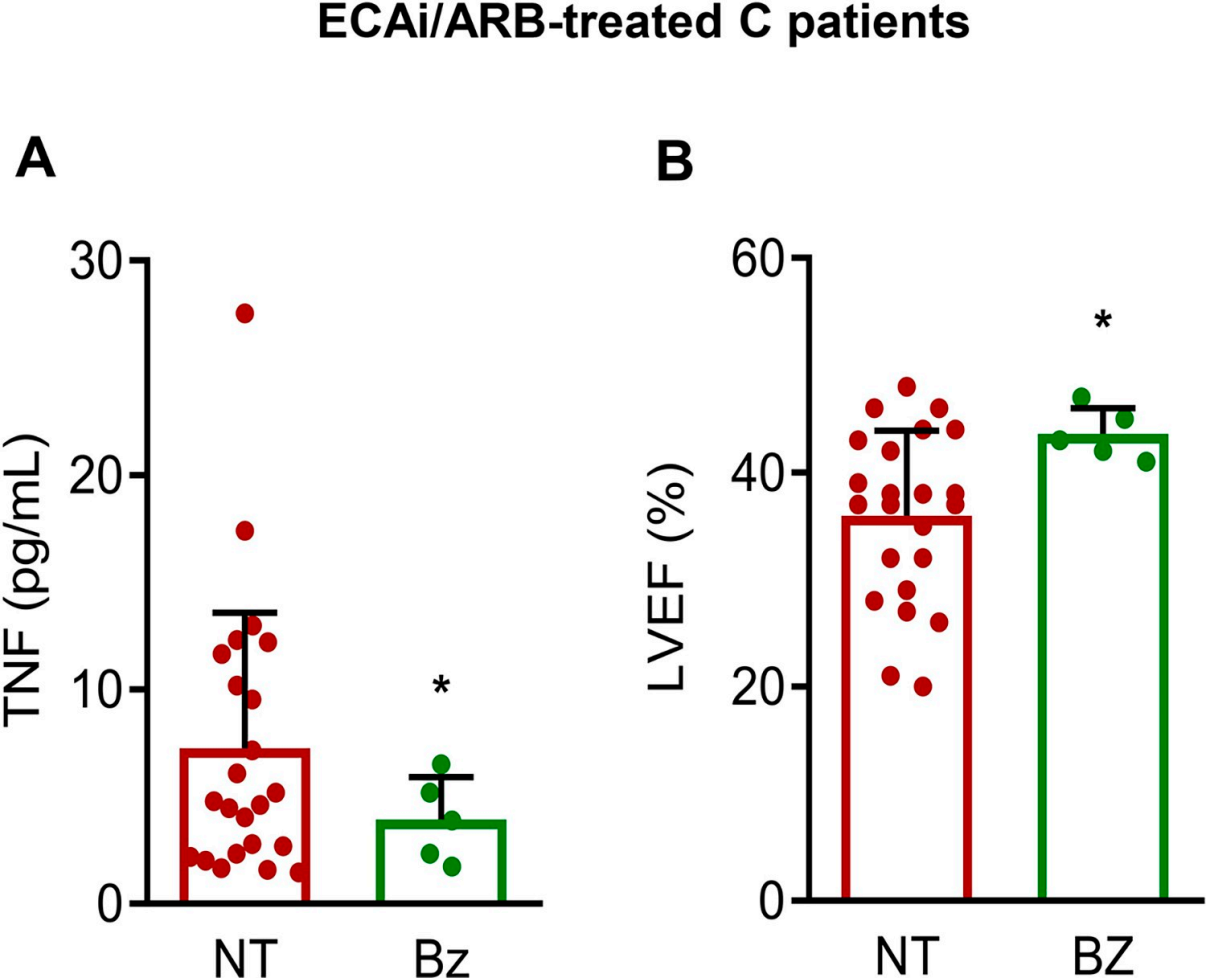
Impact of cardioprotective therapies in prior benznidazole-treated C patients. Cross-sectional observational study of groups of C patients (severe Chagas’ heart disease) administered with ACE inhibitors (ACEi) / angiotensin receptor 1 blockers (ARB) that were not treated (NT) or received benznidazole (Bz) therapy years before. **A**. TNF concentrations in serum (pg/mL). **B.** Left ventricular ejection fraction (LVEF%; Simpson’s method). ACEi/ARB (n = 23); Bz before ACEi/ARB (n = 5). The two-groups difference was analyzed with Student *t*-test. Each dot represents a patient. Data show means ± SD. *, *p* < 0.05.

Lastly, we asked about the possibility of impacting TNF serum levels and LVEF% using therapies in the group of C patients, all treated with ACEi/ARB medications. The pharmacotherapy with ACEi/ARB, used for 5 to 10 years before the inclusion of the patient in the present study, was prescribed according to clinical abnormalities [47]. Although the number of patients fulfilling the criteria of analysis was limited, our data show that TNF levels were reduced (**Fig 3A**) and LVEF% were increased (**Fig 3B**) in the group of C patients that received benznidazole therapy years before the ACEi/ARB treatment, compared to the C patients only treated with cardioprotective pharmacotherapy.

## Discussion

In a case-control study of a group of patients residing in the State of Pernambuco, Northeast region of Brazil, we provided evidence of no association of *ACE* I/D and *AGTR1* polymorphisms with susceptibility and severity of Chagas’ heart disease. However, considering the initial borderline associations, the frequencies of *ACE* I/D genotypes in A, B1, and C groups, the relation of *ACE* DD and DI and *AGTR1* rs5186 +1166 AA genotypes with reduced LVEF, and the progressive nature of Chagas’ heart disease, we consider that the *ACE* and *AGTR1* genes are candidates to be explored in precision medicine. Although with a limited number of patients, a cross-sectional observational study exposed that therapy with benznidazole and ACEi/ARB may reduce TNF serum levels and improve clinical outcome, opening a perspective of pharmacotherapy to be explored in prospective studies.

Socioeconomic indicators, such as low monthly income and schooling, revealed that the patients in the studied cohort of Northeast Brazil represent the archetypal population exposed to *T*. *cruzi* infection in Latin America. Thus, throughout their lives, they remain in a poverty-promoting situation and are vulnerable to neglected tropical diseases, conditions that are among the top threats to global health [52,53]. Most of our patients were over 45 years old, which can be explained by the decrease in transmissions by vectors and blood transfusions achieved almost two decades ago, and improved health care in Brazil [4]. Clinical evaluation showed that LVEF was preserved and similar in group A (non-cardiac form) and in group B1 (mild CARD), while HFrEF was found in C patients, supporting that an essential precondition for staging the severity of Chagas’ heart disease has been met [4,10]. In addition, the intervening variables were typical of most groups of CD patients residing in Latin America and undergoing clinical follow-up, including frequencies of gender, age, previous use of trypanocidal drugs, and prescription of cardioprotective medications [54,55]. Together, these data allowed testing of the *ACE* I/D and *AGTR1* polymorphisms as risk factors for susceptibility to Chagas’ heart disease and progression to severe CARD with HFrEF in this population.

Our primary findings supported that the DD genotype of the *ACE* I/D polymorphism was associated with progression to HF. However, after adjusting for gender, age, and trypanocidal treatment, features proposed to influence the outcome of CD [4], we found only a borderline association of *ACE* D allele with susceptibility to severe CARD, which was lost after adjustments for multiple comparisons. The observed trend reinforces the need to replicate these studies in other populations, as recommended in genetic studies involving patients with CD [12]. Despite the limited sample size and the lack of statistical support, previous study in a Venezuelan CD cohort suggested that the progression of chagasic cardiomyopathy was unrelated to *ACE* I/D polymorphism [32]. Further, in a Brazilian cohort the *ACE* I/D polymorphism was not useful to detect a relationship with HF secondary to CD [33]. Conversely, our previous study demonstrated that *ACE* DD and DI genotypes were more prevalent in CD patients with HF [16]. Consistent with these findings, regardless of the clinical classification of our patients, compared to the carriers of *ACE* II genotype D carriers exhibited a worse LVEF, which is an important prognostic factor for Chagas’ heart disease [10,12]. The influence of *ACE* DD and DI genotypes, compared to II genotype, on left ventricular remodeling has been reported [56]. In patients carrying the *ACE* II genotype, the reduced risk of developing chronic diseases may be linked to an AluYa5-controlled *ACE* gene expression [57]. Further, the *ACE* D allele may be a risk factor for the development of HF with left ventricular dysfunction after myocardial infarction [58]. Indeed, in a Brazilian cohort of patients with ischemic HF the presence of the *ACE* D allele has been associated with worsening left ventricular dysfunction [32]. In different populations, the DD genotype has been associated with phenotypes such as increased ACE activity and angiotensin II concentrations in serum [23,26,27], and enhanced ACE activity in heart tissue [59]. Further, the genotypes/phenotypes of *ACE* I/D have been related to the regulation of inflammation and tissue damage, which, in turn, are linked to cardiovascular diseases such as stroke and inflammatory heart diseases [20–22]. These findings helped predict that the *ACE* D allele is associated with increased RAS activity pathologies [60]. Indeed, higher cardiac weight was associated with the *ACE* DD genotype [61]. Moreover, a meta-analysis indicated that the *ACE* D allele is associated with an increased risk of ischemic stroke in Asians but with a borderline association for Caucasians [62]. Therefore, in CD patients, *ACE* I/D genotyping as a precision medicine tool may allow prospective study aimed at identifying other risk factors, including the risk of stroke, a critical manifestation of CD [10]. Thus, our present study sets the stage for a future study of this cohort of CD patients to elucidate the involvement of the *ACE* I/D genotypes in heart disease and stroke outcomes.

AT_1_R receptors mediate the most damaging actions of angiotensin II, including processes of vascular hypertrophy, sodium retention, cardiac remodeling, hypertension, and fibrogenesis [35]. Here, for the first time, we evaluated the association of *AGTR1* polymorphisms in the development of CARD and progression to HF in CD patients. The *AGTR1* rs5186 +1166 AC genotype showed a borderline association with protection against CARD, which was lost after adjustments for multiple comparisons. Interestingly, the *AGTR1* rs5186 +1166 AC genotype was related to better LVEF, regardless of the clinical stage. AT_1_R mRNA and protein expression are higher for carriers of the *AGTR1* +1166 CC genotype than the AC and AA genotypes [34]. Further, increased serum ACE activity and peak of cardiac troponin 1 levels, a biomarker of tissue injury, have been associated with the +1166 CC genotype in patients with acute myocardial infarction [63]. Also, an increased risk of stroke has been observed in patients with *AGTR1* +1166 CC and AC genotypes, compared to AA genotype [64]. Here, we did not identify CC homozygotes, and AC heterozygotes showed better LVEF than AA homozygotes, raising the possibility of a protective role for rs5186 +1166 AC genotype against severe CARD. In non-infectious cardiomyopathy, the CC genotype was more prevalent in patients with heart dysfunction compared to patients with preserved function [35] and was associated with lower LVEF compared to A allele carriers [65]. Thus, our results are intriguing and must be challenged in other cohorts. Our data support that the *AGTR1* SNP rs5182 C>T, rs275653 -119C>T and rs2131127 A>G were not risk factors for susceptibility to CARD or progression to severe CARD with HF. In Mexican individuals, the rs5182 CC genotype was correlated with increased blood pressure, and the risk of developing hypertension and stroke [37]. A recent study showed that the *AGTR1* rs275653 AA genotype reduced the risk of small artery occlusion strokes, although it did not affect AT_1_R expression levels [36]. In our study, no included patients had a registered previous stroke event. Thus, a longitudinal study will allow us to record this and other clinical outcomes in the present cohort. Interestingly, the rs275653/rs2131127/rs5186/rs5182 T/A/C/T haplotype was protective against progression to CARD, a condition not previously described in the literature. Nevertheless, the low frequency of this haplotype in our studied cohort does not allow for any epidemiological impact on the scenario of Chagas’ heart disease. However, it may represent an individual benefit for haplotype carriers, a condition to be further confirmed.

In a recent meta-analysis involving more than 20 thousand individuals, the *ACE* I/D polymorphism was mapped in Brazil, showing that 34% were DD, 48% DI and 18% II, and found no association with clinical conditions such as age-related, metabolic, and non-infectious heart disorders [66]. Notably, in our study the frequencies of these genotypes were dissimilar among the stage A, B1 and C groups of CD patients. Moreover, patients with the *ACE* DD and DI genotypes had lower LVEF than those with the II genotype, regardless of the clinical outcome. The *ACE* D allele has been related to the severity of cardiomyopathies of various etiologies [20,30,67]. The *ACE* DD genotype has been associated with an increased risk of HF and mortality [26] and considered a predictor of death in idiopathic HF [22], emphasizing the importance of DD genotype as a factor influencing survival in HF patients. Therefore, CD patient carriers of *ACE* D allele and, mainly the DD genotype should receive more attention and potentially be considered for early pharmacotherapy to hamper the progress of CARD to HF. In non-infectious conditions pharmacotherapy can attenuate the harmful effects of DD and DI genotypes. In untreated hypertensive patients, the *ACE* D allele, with a 192% higher risk compared to homozygous II, behaved as a marker of left ventricular hypertrophy, a condition that may be opposed by antihypertensive treatment [68]. Further, in patients with systolic HF and treated with ACEi, the *ACE* D allele was associated with an unfavorable outcome only in the group not treated with beta-blockers [69]. Thus, we attempted to explore the influence of registered treatments on clinical outcomes in the studied group of CD patients. The therapeutic response to ACEi and AT_1_R blockers has shown beneficial effects with reduced morbidity and mortality associated with CD [4,10,12,47,70]. In our studied population, 88% of stage C patients were prescribed drugs that interfere with the neurohormonal system (ACEi/ARB/diuretic/beta-blockers). Therefore, the influence of the *ACE* I/D and *AGTR1* polymorphisms on the progression of Chagas’ heart disease could have been masked using cardioprotective drugs, as ACEi/ARB were also prescribed to patients staged as B1 and A. As in other heart diseases, the treatment of HF secondary to Chagas cardiomyopathy is based on the combination of three types of drugs: diuretics, ACEi/ARB, and adrenergic blockers [10,47]. Randomized clinical trials and some retrospective studies in patients with non-infectious HFrEF demonstrate the benefits of treatment with ACEi and beta-blockers, reducing morbidity and mortality [12]. Although the evidence found in these studies is not directly linked to Chagas’ heart disease, these drugs are strongly recommended for patients with HF and LVEF ≤ 40% [10,12]. In our cohort, according to clinical abnormalities and cardiac status, drugs such as ACEi (mainly captopril, enalapril) or ARBs (mainly losartan) were prescribed for patients in group C (56.1%), B1 (23.6%) and A (13.8%). In addition, in a long-term follow-up study, specific treatment with the trypanocidal drug benznidazole was associated with a decrease in the incidence of progression from indeterminate to CARD, as well as a reduction of the risk of cardiovascular events [55]. It is important to note that benznidazole was used in patients in stage C (12.1%), B1 (10.6%), and A (32.1%). With this in mind, we envision a prospective study to understand the impact of these treatments on our CD patients.

Pro-inflammatory cytokines, such as TNF, are released shortly after *T*. *cruzi* invasion by phagocytic cells, activated lymphocytes, and other cells. This leads to chemokine production and cellular activation, enhancing the immune response involved in parasite control but also in the inflammatory response associated with tissue injury [45,71]. Individuals with chronic CD present higher TNF levels compared to non-infected individuals [12,14,46]. Elevated TNF serum levels in patients with Chagas’ heart disease have been related to the worsening of cardiac function [43,44,72]. In preclinical models, TNF plays a role in HF pathophysiology [73]. Further, in chronically *T*. *cruzi*-infected mice, TNF is associated with cardiac lesions and reduction of LVEF [74]. Here, TNF serum levels were adopted as a fingerprint of the systemic inflammatory profile described in cardiopathic CD patients [46,51]. Corroborating previous data [14], the subgroup of CD patients with severe heart disease (C group) had increased TNF serum levels when compared to patients without heart disease (A group). Interestingly, C patients treated with benznidazole years before the inclusion in our study showed TNF serum levels resembling A group patients. However, benznidazole therapy did not impact cardiac dysfunction, corroborating previous data analyzing patients 5 years post-therapy [75]. In B1 and C groups of patients, *ACE* D carriers showed higher TNF serum levels than II genotype patients, while TNF serum concentrations were not affected by *AGTR1* polymorphism, suggesting an influence of the *ACE* D/I polymorphism in inflammatory pathways in CD patients undergoing CARD. These data led us to analyze the impact of treatments interfering in the RAS axis on TNF levels and LVEF as fingerprints of immunological and clinical traits. This initial cross-sectional observational analysis supports that TNF serum levels were reduced in group C of patients who received benznidazole years before starting the treatment with ACEi/ARB, compared to C patients only treated with ACEi/ARB. Most of the C patients received only cardioprotective therapies. Moreover, the treatments (benznidazole therapy years before ACEi/ARB administration) beneficially affected the LVEF. Although limited in the number of patients, this initial observational study suggests that the sequential therapies may have favorable effects on Chagas’ heart disease. Thus, a prospective study of these patients (mainly the B1 patients), replication of this observational study, and a planned clinical trial in different populations may challenge this idea.

### Potential clinical implications

Collectively, our data support that genotyping *AGTR1* and, particularly, *ACE* I/D may add pivotal information concerning precision medicine and tailoring of cardioprotective treatments to be prescribed to CD patients, particularly to the ones with mild CARD. Further, considering the association of inflammatory profile with disease severity [43,44,51,72], our preliminary data supporting reduced TNF levels in patients that received benznidazole, a medicament efficient in reducing parasite load [75], add information on other beneficial action of this medication in chronic CD patients.

### Limitations

One limitation of most of the studies of gene polymorphisms in CD is the sample size and limited analysis of a specific cohort. We also consider these feature limitations in our study, in which we presented borderline association results lost after adjustments for multiple comparisons. However, we highlight that the sample evaluated in the present work represents a population that, although showing socioeconomic similarities, is geographically distinct from previously published literature in CD and genetic association studies regarding *ACE* and *ATGR1* gene polymorphisms. Further, this cross-sectional observational study supports that *ACE* D carriers show a more prominent systemic inflammatory profile than II carriers, thus opening an avenue to be challenged regarding the progression of Chagas’ heart disease and other clinical traits, such as stroke, that deserves a prospective study in this cohort as well as replication study in other populations, aiming, particularly, the identification of *ACE* D carriers. Although with the limitations of a sample size and the nature of a cross-sectional observational study, the obtained data instigated more robust clinical trials aiming to establish biomarkers (being TNF a mere fingerprint) to predict better prognosis after benznidazole therapy. One should keep in mind that benznidazole therapy may contribute to the control of the parasite load but also to reduce the systemic inflammation profile, conditions that may contribute to improve prognosis. Lastly, our limitations should not compromise the idea of precision medicine-based precocious administration of ACEi/ARB (and other cardioprotective drugs) to patients with mild CARD, a pharmacotherapy scheme that may add quality to our patients’ life.

## Supporting information

S1 Table Individual epidemiological, clinical classification and raw genotyping data of the 402 evaluated Chagas disease patients. (PDF)

S2 Table Individual values in a group of data used to generate graphs. (XLSX)

S3 Table Details of the TaqMan assays used for *AGTR1* SNP genotyping. (DOCX)

S1 Fig Linkage disequilibrium analysis for the tested *ATGR1* polymorphisms and evaluating the Chagas disease patients recruited for the present study. Values shown in each box and the intensity of shading are proportional to r^2^.
(TIF)

S2 Fig Effects of *ACE* and *ATGR1* polymorphisms in TNF serum levels. **A.** TNF serum concentrations (pg/mL) are shown in B1 group patients (mild Chagas’ heart disease) with *ACE* DD (n = 11), DI (n = 14) and II (n = 10) genotypes. **B**. TNF serum concentrations (pg/mL) in are shown B1 group patients classified as *ACE* D allele carriers compared to II genotype. **C**. TNF serum concentrations (pg/mL) in C group patients regarding the *ATGR1* rs5186 +1166 AA (n = 30) and AC (n = 14) genotypes. Two-groups difference was analyzed with Student *t*-test. For data composed of more than two groups, the difference between groups was analyzed using the parametric one-way ANOVA test, corrected with Turkey post hoc test with multiple comparisons. Each dot represents a patient. Data show means ± SD. *, *p* < 0.05.
(TIF)

S3 Fig Impact of benznidazole treatment on TNF serum levels and left ventricular ejection fraction. Cross-sectional observational study of groups A (non-cardiopathic) and C (severe Chagas’ heart disease) patients not-treated (NT) or administered with benznidazole (Bz) 1–20 years prior to inclusion in this study. **A**. TNF concentrations (pg/mL) in serum of A (n = 64, 27) and C (n = 47, 10) patients. **B.** Left ventricular ejection fraction (%; Simpson’s method) in A (n = 61, 23) and C (n = 43, 11) patients. For data composed of more than two groups, the difference between groups was analyzed using the parametric one-way ANOVA test, corrected with Turkey post hoc test with multiple comparisons. Each dot represents a patient. Data show means ± SD. *, *p* < 0.05, ***, *p* < 0.001.
(TIF)
