# Supplementary material for: Influence of angiotensin II type 1 receptors and angiotensin-converting enzyme I/D gene polymorphisms on the progression of Chagas’ heart disease in a Brazilian cohort: Impact of therapy on clinical outcomes
Source: PLoS Negl Trop Dis. 2024 Nov 26;18(11):e0012703. doi: 10.1371/journal.pntd.0012703 (PMC11630595; doi:10.1371/journal.pntd.0012703)
Supplement: S1 Table — (PDF) [file pntd.0012703.s001.pdf]

**Influence of angiotensin II type 1 receptors and angiotensin-converting enzyme I/D gene polymorphisms on the progression of Chagas' heart disease in a Brazilian cohort: impact of therapy on clinical outcomes**

**Running Title:** *AGTR1* and *ACE* I/D polymorphisms in Chagas' heart disease


Thayse do E.S. Protásio da Silva[1*], Lucia E. Alvarado-Arnez[1,2,#,*], Angelica M. Batista[1*], Silvia M.M. Alves[1,,3,4*], Gloria Melo[3], Cristina V. Carrazzone[illegible] Isabelle de Oliveira Moraes[2,##,] Antonio G. Pacheco[5], Camila Sarteschi[3], Milton Ozório Moraes[2§], Wilson Oliveira Jr[3], Joseli Lannes-Vieira[1]

1 Laboratório de Biologia das Interações, Instituto Oswaldo Cruz/Fiocruz, Rio de Janeiro, Brazil

2 Laboratório de Hanseníase, Instituto Oswaldo Cruz/Fiocruz, Rio de Janeiro, Brazil

3 Ambulatório de Doença de Chagas e Insuficiência Cardíaca do Pronto Socorro Cardiológico de Pernambuco (PROCAPE)/UPE, Pernambuco, Brazil

4 Instituto do Coração (InCor), Escola de Medicina, Universidade de São Paulo, Brazil

5 Programa de Computação Científica, Fiocruz, Rio de Janeiro

* These authors contribute equally to this work

# Current affiliation: Coordinación Nacional de Investigación, Universidad Privada Franz Tamayo (UNIFRANZ), La Paz, Bolivia

## Current affiliation: Laboratório de Epidemiologia de Malformações Congênitas - Instituto Oswaldo Cruz/Fiocruz

§ in memoriam


| | | | | | | | | | | | | ***ATGR1*** | | | | |
|---|---|---|---|---|---|---|---|---|---|---|---|---|---|---|---|---|
| **ID** | Gender | Age strata | Ethnicity | Monthly income | Education | Region | Stage | Benz | BB | ACEi/ARB | Spirono | rs5186 | rs5182 | rs275653 | rs2131127 | ***ACE*** I/D |
| **BR 1** | F | >45 | Mest | 1 | 4 | NA | B1 | N | N | N | N | AA | CC | CC | AA | II |
| **BR 2** | M | >45 | Mest | 1 | 4 | NA | B1 | N | N | N | N | AA | CT | CC | GG | II |
| **BR 3** | F | ≤45 | Black | 1 | >4 | Mata | B1 | Y | Y | N | N | NA | NA | NA | NA | II |
| **BR 4** | M | >45 | White | 2-4 | >4 | Mata | C | Y | Y | Y | Y | AA | NA | NA | NA | DI |
| **BR 5** | F | >45 | NA | 1 | 4 | Mata | B1 | N | N | Y | N | AC | TT | CC | GG | DI |
| **BR 6** | M | >45 | Mest | 2-4 | 4 | Mata | B1 | Y | Y | Y | N | AA | TT | CC | GA | DI |
| **BR 7** | M | ≤45 | Black | 1 | >4 | NA | B1 | N | N | N | N | AC | TT | CT | GA | II |
| **BR 8** | F | >45 | NA | 1 | 4 | Mata | B1 | N | N | N | N | AA | TT | CC | GA | DI |
| **BR 9** | M | ≤45 | Mest | 1 | 4 | Agreste | C | N | N | Y | Y | AA | CT | CC | GA | DI |
| **BR 10** | M | >45 | Black | 1 | 4 | RM | C | N | Y | Y | Y | AA | TT | CT | GG | DD |
| **BR 11** | F | >45 | White | 1 | 4 | other | C | N | N | N | N | AC | TT | CC | GA | DI |

| | | | | | | | | | | | | | | | | |
|---|---|---|---|---|---|---|---|---|---|---|---|---|---|---|---|---|
| **BR 12** | F | >45 | Mest | 1 | 4 | Mata | B1 | N | N | N | N | AA | CT | CC | GA | DI |
| **BR 13** | F | >45 | Mest | 2-4 | 4 | Agreste | B1 | N | N | N | N | AA | CT | CT | AA | DI |
| **BR 14** | M | >45 | Mest | 2-4 | 4 | Mata | A | Y | Y | N | N | AC | CT | CT | AA | II |
| **BR 15** | F | >45 | NA | NA | NA | NA | C | N | Y | Y | N | AC | TT | CC | GA | DD |
| **BR 16** | F | >45 | Mest | 1 | 4 | Agreste | C | N | N | N | N | AA | CC | CC | GA | DD |
| **BR 17** | F | ≤45 | Mest | >5 | 4 | Mata | B1 | N | N | N | N | AC | CT | CT | AA | DI |
| **BR 18** | F | ≤45 | Black | 1 | 4 | Mata | A | N | N | N | N | AC | TT | CT | GA | DI |
| **BR 19** | F | >45 | NA | 1 | 4 | Mata | B1 | N | N | N | N | AC | TT | CC | GG | DD |
| **BR 20** | F | ≤45 | Mest | NA | 4 | Sertão | B1 | N | N | N | N | AC | CT | CT | GA | DI |
| **BR 21** | M | >45 | Mest | 2-4 | 4 | Mata | B1 | N | N | N | N | AC | TT | CT | GA | DD |
| **BR 22** | F | >45 | Mest | NA | 4 | Agreste | B1 | N | N | Y | N | AC | TT | CC | GG | DI |
| **BR 23** | M | >45 | Mest | 1 | 4 | Mata | C | N | Y | Y | N | AC | CT | CC | GA | DI |
| **BR 24** | F | >45 | NA | >5 | >4 | Sertão | B1 | Y | N | N | N | AC | TT | CC | GA | II |
| **BR 25** | F | >45 | White | 1 | >4 | Mata | B1 | N | N | N | N | AC | CT | CC | GA | DI |
| **BR 26** | F | >45 | NA | 1 | 4 | other | A | N | N | N | N | AC | TT | CT | GA | DD |
| **BR 27** | F | >45 | Mest | 1 | 4 | Agreste | C | N | N | N | N | AA | CT | CC | AA | II |
| **BR 28** | F | >45 | Mest | <2 | >4 | Sertão | B1 | N | N | N | N | AA | TT | CC | GG | DI |
| **BR 29** | M | >45 | Mest | 1 | 4 | Mata | C | N | N | N | N | NA | TT | CT | AA | DD |
| **BR 30** | F | >45 | White | 1 | 4 | Sertão | B1 | Y | N | N | N | AA | CT | CC | GA | DI |
| **BR 31** | F | >45 | NA | 1 | >4 | Mata | B1 | N | N | N | N | AC | CT | CT | GA | DI |
| **BR 32** | M | >45 | Black | 1 | 4 | other | C | N | Y | Y | N | AA | CC | CC | AA | DI |
| **BR 33** | M | ≤45 | Mest | 1 | >4 | Sertão | A | N | N | N | N | AC | TT | CC | GA | II |
| **BR 34** | F | >45 | Mest | 1 | >4 | other | B1 | N | N | N | N | AA | CC | CC | GA | II |
| **BR 35** | F | >45 | Mest | 1 | 4 | Mata | B1 | N | N | Y | N | AA | CC | CC | AA | II |
| **BR 36** | F | >45 | Mest | 1 | 4 | Mata | B1 | N | N | N | N | AA | CT | CC | GA | DI |
| **BR 37** | F | >45 | Black | 1 | 4 | Mata | B1 | N | N | Y | N | AA | CT | CC | GA | DI |
| **BR 38** | F | >45 | Mest | <1 | 4 | Mata | B1 | N | N | Y | N | AC | CT | CT | AA | DI |
| **BR 39** | F | >45 | White | 1 | 4 | Mata | B1 | N | N | N | N | AC | TT | CC | GA | DI |
| **BR 40** | M | >45 | Mest | NA | 4 | Mata | C | N | N | N | N | AC | TT | CC | GG | DI |
| **BR 41** | F | >45 | Mest | 1 | 4 | Mata | B1 | N | N | N | N | AA | CC | CC | GG | II |
| **BR 42** | M | >45 | Black | <1 | 4 | Mata | B1 | N | N | N | N | AA | TT | CT | GA | DD |
| **BR 43** | F | >45 | White | 2-4 | >4 | Mata | B1 | N | N | N | N | AC | CT | CT | AA | II |
| **BR 44** | F | >45 | White | 1 | 4 | Mata | B1 | N | N | N | N | AA | TT | TT | GG | DD |

| | | | | | | | | | | | | | | | | |
|---|---|---|---|---|---|---|---|---|---|---|---|---|---|---|---|---|
| **BR 45** | M | ≤45 | Mest | 1 | 4 | other | B1 | N | N | N | N | AC | CT | CC | GG | DD |
| **BR 46** | F | >45 | NA | <1 | 4 | Sertão | B1 | N | Y | Y | N | AA | CT | CC | GA | DI |
| **BR 47** | F | >45 | Mest | 1 | 4 | Sertão | B1 | N | N | N | N | AC | CT | CT | GA | DI |
| **BR 48** | M | ≤45 | Black | 1 | >4 | RM | A | N | N | N | N | AA | CT | CC | GG | II |
| **BR 49** | F | ≤45 | NA | 1 | >4 | Sertão | B1 | Y | N | N | N | AA | CT | CC | GA | DD |
| **BR 50** | F | >45 | Mest | 1 | >4 | NA | C | N | Y | Y | N | AA | CT | CC | GA | DD |
| **BR 51** | F | >45 | Mest | 1 | 4 | Mata | B1 | N | N | N | N | AA | CT | TT | GA | DI |
| **BR 52** | F | >45 | Black | >5 | 4 | Agreste | C | N | N | N | Y | AA | TT | CC | GG | DI |
| **BR 53** | M | >45 | Mest | 1 | 4 | Mata | C | N | N | N | N | AA | CT | TT | AA | DD |
| **BR 54** | F | >45 | Mest | 1 | 4 | Mata | B1 | N | N | N | N | AA | TT | CC | GG | DD |
| **BR 55** | M | >45 | Black | 1 | 4 | Mata | C | N | Y | Y | Y | AA | CT | CT | AA | DD |
| **BR 56** | M | >45 | NA | 1 | 4 | Agreste | B1 | N | N | N | N | AA | TT | CC | AA | DI |
| **BR 57** | F | >45 | Mest | 1 | 4 | Agreste | A | N | N | N | N | AC | TT | CT | AA | DI |
| **BR 58** | F | >45 | NA | 1 | 4 | Sertão | A | Y | Y | N | Y | AC | CT | CC | GA | DD |
| **BR 59** | M | >45 | Mest | 1 | >4 | Agreste | A | N | N | N | N | AC | CT | CC | AA | DI |
| **BR 60** | F | >45 | Mest | 1-2 | >4 | Sertão | B1 | N | N | N | N | AC | TT | CC | AA | DI |
| **BR 61** | M | >45 | Mest | 2-4 | >4 | Sertão | C | N | N | N | N | AA | CT | CT | AA | DI |
| **BR 62** | M | >45 | Mest | 1 | 4 | Sertão | B1 | N | N | N | N | AA | CT | CT | GA | DI |
| **BR 63** | F | >45 | Mest | 1 | 4 | Mata | C | N | N | N | N | AA | CT | CT | GA | DD |
| **BR 64** | F | >45 | Mest | <1 | 4 | Sertão | C | N | N | N | N | AA | CT | CT | GA | DD |
| **BR 65** | F | >45 | Mest | 1 | 4 | Sertão | C | Y | N | N | N | AA | CC | CC | AA | II |
| **BR 66** | F | >45 | NA | 1 | 4 | NA | B1 | N | N | N | N | NA | TT | CT | AA | DD |
| **BR 67** | F | >45 | NA | NA | NA | NA | C | N | N | N | N | AA | CC | CT | GA | DI |
| **BR 68** | F | >45 | Mest | 1 | 4 | Sertão | B1 | N | N | N | N | AA | TT | CT | GA | DI |
| **BR 69** | F | >45 | White | 1 | 4 | Sertão | C | N | N | N | N | AA | CC | CC | GA | II |
| **BR 70** | F | >45 | White | 1 | >4 | Mata | B1 | N | N | N | N | AC | CT | CT | AA | II |
| **BR 71** | M | >45 | White | 1 | 4 | Agreste | A | N | N | N | N | AA | CC | CC | GG | DI |
| **BR 72** | F | >45 | Mest | 1 | 4 | Mata | B1 | N | N | N | N | AA | CT | CT | GG | DI |
| **BR 73** | M | >45 | Black | >5 | 4 | Sertão | C | Y | Y | Y | N | AA | TT | CC | GG | DD |
| **BR 74** | F | >45 | White | 1 | 4 | Mata | A | N | N | N | N | AC | CT | CC | AA | DD |
| **BR 75** | F | >45 | Black | >5 | 4 | Mata | C | N | Y | Y | Y | AA | TT | CT | AA | DI |
| **BR 76** | M | ≤45 | Mest | 1 | 4 | Mata | C | N | Y | Y | N | AA | CT | CC | GA | DD |
| **BR 77** | F | ≤45 | Mest | 1 | 4 | Agreste | B1 | Y | Y | N | N | AA | CT | CT | GA | DI |

| | | | | | | | | | | | | | | | | |
|---|---|---|---|---|---|---|---|---|---|---|---|---|---|---|---|---|
| **BR 78** | F | >45 | Mest | NA | 4 | Agreste | C | N | N | N | N | NA | NA | NA | NA | DI |
| **BR 79** | F | >45 | Mest | 1 | 4 | Mata | B1 | Y | N | N | N | AC | CT | CT | AA | DD |
| **BR 80** | F | >45 | Mest | 1 | 4 | Agreste | B1 | N | N | Y | N | AC | CT | CC | GA | DD |
| **BR 81** | F | >45 | Mest | 1 | 4 | Mata | B1 | N | Y | Y | N | AC | TT | CC | GA | DI |
| **BR 82** | F | >45 | Mest | 1 | 4 | Mata | B1 | N | N | N | N | AC | CT | CC | GA | DI |
| **BR 83** | F | >45 | White | 2-4 | >4 | NA | B1 | Y | N | N | N | AA | CT | CC | GA | NA |
| **BR 84** | F | >45 | Mest | 1 | >4 | Sertão | C | Y | N | N | N | AA | CT | CC | GA | II |
| **BR 85** | F | >45 | Mest | NA | 4 | Mata | B1 | N | N | Y | N | AA | TT | CT | GG | II |
| **BR 86** | F | ≤45 | Mest | <1 | 4 | Mata | B1 | N | N | N | N | AA | CT | CC | GG | II |
| **BR 87** | M | >45 | NA | 1 | 4 | Mata | B1 | N | Y | N | N | AA | TT | CC | GA | DI |
| **BR 88** | M | >45 | White | >5 | 4 | Agreste | A | Y | N | Y | N | AC | CT | CC | AA | DI |
| **BR 89** | F | ≤45 | Mest | >5 | 4 | other | A | N | N | N | N | AA | CT | CC | GA | II |
| **BR 90** | F | >45 | White | NA | >4 | Agreste | B1 | N | Y | Y | N | AA | CT | CT | GA | DD |
| **BR 91** | F | >45 | White | 2 | 4 | Agreste | C | N | Y | Y | N | AA | CT | CC | GA | DI |
| **BR 92** | F | >45 | Mest | >5 | 4 | other | B1 | N | N | N | N | AC | TT | CC | AA | DD |
| **BR 93** | F | >45 | Mest | 1 | 4 | Mata | B1 | N | N | N | N | AA | TT | CT | GA | DD |
| **BR 94** | F | ≤45 | Mest | NA | 4 | Mata | C | N | N | N | N | AA | TT | CT | AA | DI |
| **BR 95** | F | >45 | Mest | 1 | 4 | other | C | N | N | Y | N | AC | TT | CC | GA | DD |
| **BR 96** | M | >45 | White | 1 | 4 | Sertão | A | N | N | N | N | AC | TT | CC | AA | DD |
| **BR 97** | F | >45 | Black | NA | >4 | Mata | B1 | N | Y | N | N | AA | CT | CC | GA | DI |
| **BR 98** | F | >45 | Mest | 1 | 4 | Sertão | B1 | N | Y | Y | N | AC | TT | CC | GG | DD |
| **BR 99** | M | >45 | White | 1 | 4 | other | C | N | Y | Y | N | AA | CT | CT | GA | II |
| **BR 100** | F | >45 | Mest | NA | 4 | NA | B1 | N | N | N | N | AC | TT | CT | GA | II |
| **BR 101** | M | >45 | Mest | 1 | 4 | Mata | B1 | N | N | Y | N | AA | CC | CC | AA | DI |
| **BR 102** | F | >45 | Mest | >5 | 4 | other | A | N | N | N | N | AC | TT | CT | AA | DI |
| **BR 103** | F | >45 | White | 1 | 4 | other | B1 | N | N | N | N | AC | TT | CC | GA | DD |
| **BR 104** | F | >45 | Mest | 1 | 4 | Mata | C | N | N | N | N | AA | CT | CT | GA | DD |
| **BR 105** | F | >45 | Mest | >5 | 4 | other | B1 | N | N | N | N | AC | CT | CC | AA | DI |
| **BR 106** | F | >45 | Mest | 1 | >4 | Mata | B1 | N | N | N | N | AC | TT | CC | GG | II |
| **BR 107** | M | ≤45 | Mest | 1 | 4 | Mata | C | N | N | N | N | AA | CT | CC | GA | DI |
| **BR 108** | M | >45 | Mest | 1 | 4 | other | A | N | N | Y | N | AA | CT | CC | AA | DD |
| **BR 109** | M | >45 | Mest | 1 | 4 | Agreste | A | N | N | N | N | AA | CT | CT | GA | DD |
| **BR 110** | F | >45 | Mest | 1 | 4 | Mata | B1 | N | N | N | N | AA | CT | CT | GA | DI |

| | | | | | | | | | | | | | | | | |
|---|---|---|---|---|---|---|---|---|---|---|---|---|---|---|---|---|
| **BR 111** | F | >45 | White | 1 | >4 | other | B1 | N | N | N | N | AA | CT | CC | GA | DI |
| **BR 112** | M | >45 | Mest | 1 | >4 | other | B1 | N | N | N | N | AA | TT | CC | GG | DI |
| **BR 113** | F | >45 | Black | 1 | 4 | Mata | B1 | N | N | N | N | AC | TT | CC | GG | DD |
| **BR 114** | F | >45 | Mest | 1 | >4 | Sertão | B1 | Y | Y | Y | N | AA | CT | CC | GA | DI |
| **BR 115** | F | >45 | White | 1 | 4 | Agreste | B1 | Y | N | N | N | AC | CT | CC | AA | II |
| **BR 116** | F | >45 | Mest | NA | 4 | Sertão | B1 | N | Y | N | N | AA | CT | CC | GA | DI |
| **BR 117** | F | ≤45 | Mest | NA | 4 | Sertão | B1 | N | N | N | N | AA | CT | CT | GA | II |
| **BR 118** | M | >45 | White | 2-4 | 4 | Sertão | A | N | Y | N | N | AA | CC | CT | GA | II |
| **BR 119** | M | >45 | Black | 1 | 4 | NA | C | N | N | Y | N | AC | CT | CT | AA | II |
| **BR 120** | M | >45 | Black | 1 | 4 | Agreste | C | Y | Y | Y | Y | AA | TT | CT | GA | DD |
| **BR 121** | M | >45 | Mest | NA | 4 | Agreste | C | N | N | Y | Y | AA | CT | CC | GA | DI |
| **BR 122** | F | ≤45 | NA | 1 | 4 | Mata | C | Y | Y | Y | Y | AC | TT | CC | GA | DI |
| **BR 123** | M | >45 | Mest | NA | 4 | NA | C | N | N | N | N | AA | CC | CC | GG | DD |
| **BR 124** | M | >45 | White | 1 | 4 | Agreste | C | N | Y | Y | N | AC | TT | TT | AA | II |
| **BR 125** | F | >45 | NA | 1 | 4 | Agreste | C | N | Y | Y | N | AC | CT | TT | AA | DI |
| **BR 126** | F | >45 | Mest | NA | 4 | Mata | B1 | N | N | Y | N | AC | TT | CC | GA | DI |
| **BR 127** | F | >45 | Mest | 1 | 4 | Mata | B1 | N | Y | N | N | AC | TT | TT | GA | II |
| **BR 128** | F | >45 | Mest | 1 | 4 | RM | B1 | N | N | Y | N | AA | CT | CT | GA | DI |
| **BR 129** | F | >45 | Mest | 1 | 4 | Other | C | Y | N | N | N | AA | CC | CC | AA | DI |
| **BR 130** | M | >45 | Mest | 1 | 4 | Mata | B1 | N | N | Y | N | AC | TT | CC | GG | II |
| **BR 131** | F | >45 | White | >5 | 4 | Sertão | A | Y | Y | N | N | AA | CC | CC | GA | DD |
| **BR 132** | F | >45 | Mest | >5 | 4 | other | B1 | N | N | Y | N | AC | TT | CT | GA | DD |
| **BR 133** | M | >45 | Mest | 1 | 4 | other | C | N | Y | Y | Y | AC | CT | CC | AA | II |
| **BR 134** | M | >45 | White | 1 | 4 | Sertão | B1 | Y | N | Y | N | AA | CT | CC | GA | DI |
| **BR 135** | F | >45 | White | >5 | 4 | Agreste | A | Y | Y | Y | N | AA | CT | CC | GG | DI |
| **BR 136** | M | >45 | NA | 1 | 4 | Mata | C | N | N | N | N | AC | TT | CT | GA | DI |
| **BR 137** | M | >45 | Mest | 1 | 4 | other | A | N | N | N | N | AA | TT | CC | GG | DI |
| **BR 138** | F | >45 | Black | >5 | 4 | other | A | N | N | N | N | AC | TT | CC | AA | DI |
| **BR 139** | F | ≤45 | Mest | >5 | 4 | Sertão | A | Y | N | N | N | AC | TT | CT | GA | DI |
| **BR 140** | F | >45 | Mest | NA | >4 | Agreste | B1 | N | N | N | N | AA | TT | CT | AA | DI |
| **BR 141** | M | >45 | NA | 1 | 4 | Agreste | C | N | Y | Y | N | AA | CT | CC | GG | DI |
| **BR 142** | F | >45 | Mest | 1 | 4 | Sertão | B1 | Y | N | N | N | AA | CC | CC | GA | DD |
| **BR 143** | F | >45 | Mest | 1 | 4 | other | B1 | N | N | N | N | AA | CT | CC | GA | DD |

| | | | | | | | | | | | | | | | | |
|---|---|---|---|---|---|---|---|---|---|---|---|---|---|---|---|---|
| **BR 144** | F | ≤45 | Mest | <1 | 4 | Agreste | B1 | N | N | N | N | AC | TT | CC | GA | DI |
| **BR 145** | F | ≤45 | Mest | 1 | 4 | other | C | N | Y | Y | Y | AA | CC | CC | GA | II |
| **BR 146** | F | >45 | Mest | 1 | 4 | Mata | B1 | Y | N | Y | N | AA | TT | CC | GG | II |
| **BR 147** | M | >45 | Mest | 1 | 4 | Other | B1 | N | N | Y | N | AA | CC | CC | GA | DI |
| **BR 148** | F | >45 | Mest | 1 | >4 | NA | C | N | Y | Y | N | AC | CT | CT | AA | II |
| **BR 149** | F | >45 | Black | 1 | 4 | Mata | B1 | N | N | Y | N | AC | TT | CC | GA | DD |
| **BR 150** | F | >45 | Mest | 2-4 | 4 | Sertão | C | N | N | N | N | AC | CT | CC | GA | DD |
| **BR 151** | M | >45 | Mest | 1 | 4 | Agreste | C | N | Y | Y | Y | AC | CT | CC | GA | DI |
| **BR 152** | F | >45 | Mest | >5 | 4 | other | C | N | Y | Y | Y | AA | CT | CC | AA | DI |
| **BR 153** | F | >45 | Mest | 1 | 4 | other | C | N | Y | Y | N | AA | TT | CT | GA | DI |
| **BR 154** | M | >45 | Mest | 1 | 4 | Agreste | C | N | N | N | N | AA | TT | CT | GA | DI |
| **BR 155** | F | >45 | White | >5 | 4 | Agreste | C | N | Y | Y | Y | AA | CC | CC | GA | DI |
| **BR 156** | F | >45 | Mest | 1 | 4 | Mata | C | N | Y | Y | Y | NA | NA | NA | NA | DD |
| **BR 157** | F | >45 | White | 1 | 4 | Mata | C | N | N | Y | N | AA | NA | NA | NA | DD |
| **BR 158** | F | >45 | Mest | 1 | 4 | Mata | C | N | Y | Y | Y | AC | TT | CC | GA | DI |
| **BR 159** | F | >45 | Mest | 1 | 4 | RM | C | N | Y | Y | Y | AA | CT | CT | GG | DD |
| **BR 160** | M | >45 | White | 1 | 4 | Mata | B1 | N | N | Y | N | AC | TT | CT | GA | DI |
| **BR 161** | M | >45 | White | 1 | >4 | Agreste | C | N | Y | Y | Y | AA | CT | CT | GA | DI |
| **BR 162** | M | >45 | Mest | 1 | 4 | Mata | C | N | Y | Y | N | AA | CT | CT | AA | DD |
| **BR 163** | F | >45 | Mest | 1 | 4 | Mata | C | N | Y | Y | N | NA | NA | NA | NA | DI |
| **BR 164** | M | >45 | Mest | 1 | 4 | Agreste | C | N | Y | Y | Y | AA | CC | CC | AA | DI |
| **BR 165** | M | >45 | Mest | 1 | 4 | Mata | C | N | Y | Y | Y | AA | CT | CC | GG | DD |
| **BR 166** | M | >45 | Mest | 1 | 4 | Agreste | C | N | N | Y | Y | AC | CT | CC | GG | DI |
| **BR 167** | M | >45 | NA | NA | NA | NA | C | N | Y | Y | Y | AA | TT | CT | GA | DI |
| **BR 168** | M | >45 | Mest | 1 | 4 | Agreste | B1 | N | N | N | N | AC | TT | CT | AA | II |
| **BR 169** | F | >45 | Black | NA | 4 | Mata | B1 | N | N | N | N | AC | TT | CT | GA | DI |
| **BR 170** | M | ≤45 | Mest | NA | 4 | RM | B1 | N | N | N | N | AC | TT | CT | GA | DD |
| **BR 171** | F | >45 | Mest | 2-4 | 4 | Sertão | B1 | N | N | N | N | AA | CC | CT | AA | DI |
| **BR 172** | M | >45 | White | NA | 4 | Agreste | A | N | N | N | N | AA | CC | CT | GA | DI |
| **BR 173** | F | >45 | Mest | 1 | NA | NA | C | N | N | N | N | AA | CT | CC | GA | DD |
| **BR 174** | M | >45 | Mest | 1 | NA | Agreste | C | N | Y | Y | Y | AA | CT | CC | GA | DI |
| **BR 175** | M | >45 | Mest | 1 | 4 | Mata | B1 | N | N | Y | N | AA | TT | CT | GA | II |
| **BR 176** | F | >45 | Mest | 1 | 4 | Sertão | B1 | N | N | Y | N | AA | CT | CC | GG | DI |

| | | | | | | | | | | | | | | | | |
|---|---|---|---|---|---|---|---|---|---|---|---|---|---|---|---|---|
| **BR 177** | F | >45 | Mest | 1 | 4 | Mata | A | N | N | Y | N | AC | TT | CC | GA | DI |
| **BR 178** | F | >45 | Mest | 1 | 4 | Mata | C | N | N | N | N | AC | CT | CT | GA | DD |
| **BR 179** | F | ≤45 | White | 1 | 4 | Mata | B1 | Y | N | N | N | AC | TT | CT | GG | DD |
| **BR 180** | F | >45 | Mest | >5 | 4 | other | A | N | N | Y | N | AA | TT | CT | GA | II |
| **BR 181** | F | >45 | Mest | 1 | 4 | Mata | B1 | N | N | N | N | AC | TT | NA | GA | DD |
| **BR 182** | F | >45 | White | 1 | 4 | Agreste | C | N | Y | Y | Y | AC | CT | CC | GA | II |
| **BR 183** | F | >45 | Mest | 1 | 4 | NA | C | N | Y | N | N | AC | TT | CT | GA | DD |
| **BR 184** | M | ≤45 | Mest | 1 | 4 | Agreste | B1 | N | N | N | N | AC | TT | CT | AA | DD |
| **BR 185** | F | >45 | Mest | 1 | 4 | other | B1 | N | N | Y | N | AA | TT | TT | GG | DI |
| **BR 186** | F | >45 | Mest | NA | NA | other | B1 | N | N | N | N | AA | CT | CC | GA | DD |
| **BR 187** | F | >45 | Mest | NA | >4 | RM | B1 | N | N | N | N | AA | CT | NA | NA | DD |
| **BR 188** | F | >45 | Mest | 1 | 4 | Agreste | B1 | N | N | N | N | AA | CC | CC | AA | DD |
| **BR 189** | F | ≤45 | Mest | 2-4 | >4 | RM | B1 | N | N | N | N | AC | CT | TT | AA | DD |
| **BR 190** | F | >45 | Mest | NA | NA | Mata | B1 | N | N | N | N | AC | TT | CT | GA | DI |
| **BR 191** | F | >45 | White | 1 | 4 | Agreste | C | N | Y | Y | Y | AC | TT | CC | GA | DI |
| **BR 192** | F | >45 | Mest | 1 | NA | Sertão | B1 | N | N | Y | N | AC | TT | CC | GA | DD |
| **BR 193** | M | >45 | Mest | 1 | 4 | Agreste | C | N | Y | Y | Y | AA | TT | CT | GA | DI |
| **BR 194** | F | >45 | White | 1 | 4 | Sertão | B1 | Y | N | N | N | AC | TT | CT | GA | DI |
| **BR 195** | F | >45 | Mest | 1 | 4 | Mata | B1 | N | N | N | N | AA | TT | CT | GA | DI |
| **BR 196** | M | >45 | Mest | 1 | 4 | Mata | B1 | N | N | Y | N | AA | CT | CC | GG | DI |
| **BR 197** | M | >45 | Mest | 1 | 4 | Agreste | B1 | N | N | N | N | AA | TT | CT | GA | DD |
| **BR 198** | F | >45 | NA | 1 | 4 | Mata | B1 | N | Y | Y | N | AA | TT | CC | NA | DD |
| **BR 199** | M | ≤45 | Black | 1-2 | 4 | RM | B1 | N | N | N | N | AA | CT | CT | GA | DI |
| **BR 200** | M | >45 | White | 1 | 4 | Mata | C | N | Y | N | Y | AA | CT | CC | GA | DI |
| **BR 201** | F | >45 | Mest | 1 | 4 | Sertão | C | N | Y | Y | Y | AA | CC | CC | GA | II |
| **BR 202** | F | >45 | White | 2-4 | 4 | Mata | B1 | N | N | Y | N | AC | TT | CT | AA | DD |
| **BR 203** | F | >45 | Mest | 1 | 4 | Mata | C | N | N | N | N | AC | TT | CT | GA | NA |
| **BR 204** | F | >45 | Mest | 1 | 4 | Sertão | C | N | Y | Y | Y | AC | TT | CC | GA | DI |
| **BR 205** | F | >45 | Mest | 1 | 4 | Agreste | B1 | N | N | Y | N | AC | TT | TT | AA | DD |
| **BR 206** | F | >45 | Mest | NA | 4 | Agreste | A | N | N | N | N | AA | CC | CT | AA | DD |
| **BR 207** | F | >45 | Black | 1 | 4 | Mata | B1 | N | N | N | N | AC | CT | CT | GA | DI |
| **BR 208** | F | >45 | Mest | NA | 4 | Mata | C | N | Y | Y | N | AC | CT | CC | GG | DD |
| **BR 209** | F | ≤45 | Mest | >5 | 4 | other | A | N | N | N | N | AA | CT | CC | GA | DD |

| | | | | | | | | | | | | | | | | |
|---|---|---|---|---|---|---|---|---|---|---|---|---|---|---|---|---|
| **BR 210** | F | >45 | Mest | 1 | 4 | Mata | B1 | N | Y | Y | N | AC | TT | CT | GA | DI |
| **BR 211** | F | >45 | White | 1 | 4 | Sertão | B1 | N | N | N | N | AA | TT | CC | GG | II |
| **BR 212** | M | ≤45 | Mest | 2-4 | 4 | Agreste | B1 | N | N | N | N | AC | TT | CT | GA | DI |
| **BR 213** | F | >45 | White | <1 | 4 | Sertão | C | N | Y | Y | Y | AA | CT | CC | GA | DI |
| **BR 214** | F | >45 | Mest | 2-4 | 4 | Mata | A | Y | Y | Y | N | AA | TT | CT | AA | DD |
| **BR 215** | F | >45 | Mest | 1 | 4 | Agreste | A | N | N | N | N | AA | CT | CT | GA | DD |
| **BR 216** | F | >45 | Mest | 1 | 4 | Mata | C | N | N | N | N | AA | CT | CC | GA | DD |
| **BR 217** | F | ≤45 | NA | >5 | >4 | RM | C | Y | Y | Y | N | AA | TT | CT | GA | DI |
| **BR 218** | F | >45 | Mest | NA | NA | Mata | B1 | N | N | N | N | AA | TT | CC | GA | DD |
| **BR 219** | M | ≤45 | NA | NA | NA | NA | C | Y | Y | Y | Y | AC | CT | CT | GA | DD |
| **BR 220** | M | >45 | Mest | NA | NA | Agreste | C | N | Y | Y | Y | AA | CT | CC | GG | DI |
| **BR 221** | F | >45 | Mest | <1 | NA | Mata | C | N | N | N | N | AC | CT | CC | GA | DD |
| **BR 222** | F | >45 | NA | 1 | NA | Mata | C | Y | N | Y | N | AA | CT | TT | AA | DD |
| **BR 223** | F | >45 | NA | 1 | 4 | Mata | C | N | Y | Y | Y | AA | CC | CC | GA | DD |
| **BR 224** | F | >45 | Mest | 1 | 4 | Mata | B1 | N | N | Y | N | AC | TT | CT | GA | DI |
| **BR 225** | F | >45 | Mest | 1 | >4 | RM | B1 | N | Y | N | N | AA | TT | CC | GG | DI |
| **BR 226** | F | >45 | Mest | 1 | 4 | Mata | B1 | N | N | N | N | AC | TT | CC | GA | DD |
| **BR 227** | F | >45 | Mest | NA | 4 | Mata | C | N | N | Y | Y | AA | TT | CC | GA | DD |
| **BR 228** | F | ≤45 | Black | >5 | 4 | Sertão | A | Y | N | N | N | AC | TT | CC | GA | DD |
| **BR 229** | F | >45 | Mest | 1 | 4 | Mata | C | N | Y | N | Y | AC | TT | CT | AA | DI |
| **BR 230** | F | >45 | Black | 1 | 4 | Mata | C | N | Y | Y | N | AA | TT | CC | GG | II |
| **BR 231** | M | >45 | Mest | 2-4 | 4 | NA | C | N | Y | Y | Y | AA | CT | CC | AA | DD |
| **BR 232** | M | >45 | Mest | 1 | 4 | Agreste | C | N | Y | Y | Y | AA | TT | CC | GG | DI |
| **BR 233** | M | >45 | White | 1 | 4 | Sertão | C | Y | Y | Y | Y | AA | CC | CC | GA | DI |
| **BR 234** | F | >45 | White | 1 | 4 | Mata | B1 | N | Y | N | N | AA | CT | CT | GA | DI |
| **BR 235** | F | >45 | Mest | 1 | 4 | Sertão | B1 | N | Y | Y | N | AC | CT | CT | GA | DD |
| **BR 236** | F | ≤45 | Mest | >5 | 4 | other | A | Y | N | N | N | AC | TT | CT | GA | II |
| **BR 237** | F | >45 | Mest | 1 | 4 | Sertão | B1 | N | Y | N | N | AA | CT | CT | AA | DD |
| **BR 238** | F | >45 | Mest | 1 | >4 | other | C | N | N | N | N | AA | CC | CT | GA | DI |
| **BR 239** | M | >45 | Mest | NA | NA | Sertão | A | N | N | N | N | AC | TT | CC | AA | DD |
| **BR 240** | F | >45 | Mest | NA | 4 | Agreste | B1 | N | N | N | N | AC | TT | CC | AA | DD |
| **BR 241** | F | >45 | White | 1 | 4 | other | A | N | N | N | N | AA | CT | CT | AA | DI |
| **BR 242** | F | >45 | NA | NA | 4 | Agreste | A | Y | N | N | N | AA | CT | CT | AA | II |

| | | | | | | | | | | | | | | | | |
|---|---|---|---|---|---|---|---|---|---|---|---|---|---|---|---|---|
| **BR 243** | M | ≤45 | White | 1 | 4 | Sertão | C | N | N | N | N | NA | CC | CC | AA | DI |
| **BR 244** | M | ≤45 | NA | NA | NA | Sertão | B1 | N | N | N | N | AC | CT | CC | GA | II |
| **BR 245** | F | >45 | NA | NA | NA | Mata | B1 | N | N | N | N | AA | TT | CC | GG | DD |
| **BR 246** | F | >45 | Mest | 2-4 | NA | Agreste | B1 | Y | N | Y | N | AA | CC | CC | AA | NA |
| **BR 247** | F | >45 | White | NA | 4 | Mata | B1 | N | N | N | N | AC | TT | CC | GG | DI |
| **BR 248** | F | >45 | White | NA | NA | Mata | B1 | N | N | N | N | AA | CT | TT | AA | DI |
| **BR 249** | F | ≤45 | Mest | 1 | >4 | Mata | B1 | N | N | N | N | AC | TT | TT | GA | DD |
| **BR 250** | F | >45 | White | 1 | >4 | Agreste | A | Y | N | N | N | AC | CT | CC | GA | DD |
| **BR 251** | F | >45 | Mest | 1 | 4 | Agreste | B1 | N | N | N | N | AA | TT | CT | GG | II |
| **BR 252** | M | ≤45 | White | NA | 4 | Sertão | B1 | N | N | N | N | AC | CT | CC | GA | II |
| **BR 253** | M | >45 | Mest | 1 | 4 | Mata | C | N | N | N | N | AA | TT | CC | GA | DD |
| **BR 254** | F | >45 | White | 1 | 4 | Agreste | C | N | Y | N | N | AC | TT | CT | AA | DD |
| **BR 255** | M | ≤45 | Mest | NA | 4 | Sertão | B1 | Y | N | Y | N | AC | TT | CT | AA | DI |
| **BR 256** | F | >45 | Mest | 1 | 4 | Mata | B1 | N | N | Y | N | AA | TT | CC | GA | DI |
| **BR 257** | M | >45 | Mest | 2-4 | >4 | other | C | N | N | N | N | AA | CT | CC | GG | DD |
| **BR 258** | M | >45 | Mest | 1 | 4 | Mata | B1 | N | N | N | N | AA | CC | CC | GA | DI |
| **BR 259** | F | >45 | Mest | >5 | >4 | NA | A | Y | N | N | N | AA | CT | CC | GA | DI |
| **BR 260** | F | ≤45 | Black | 1 | 4 | RM | C | N | Y | Y | Y | AA | CT | CT | GG | II |
| **BR 261** | F | >45 | Mest | 2-4 | 4 | NA | B1 | N | N | N | N | AA | CC | CC | NA | NA |
| **BR 262** | F | >45 | Mest | NA | 4 | Mata | C | N | N | N | N | AC | TT | CT | GG | DD |
| **BR 263** | F | >45 | Mest | NA | 4 | Mata | C | N | N | N | N | AA | CC | CC | GA | DI |
| **BR 264** | M | >45 | Mest | >5 | >4 | other | A | N | N | N | N | AA | CT | CT | GA | II |
| **BR 265** | M | >45 | Black | 1 | 4 | other | A | Y | N | Y | N | AC | CT | CC | AA | DI |
| **BR 266** | M | >45 | Mest | 1 | 4 | Agreste | A | N | N | N | N | AA | CT | CT | AA | II |
| **BR 267** | F | ≤45 | Mest | 1 | >4 | Mata | B1 | N | N | N | N | AA | CT | CC | GG | NA |
| **BR 268** | F | >45 | Mest | 1 | 4 | Mata | C | N | N | N | N | AC | TT | CC | AA | NA |
| **BR 269** | M | >45 | Mest | 1 | 4 | Mata | C | N | Y | Y | Y | AA | CT | CC | GG | DD |
| **BR 270** | F | >45 | White | 1 | 4 | Mata | B1 | N | Y | Y | N | AA | CT | CT | GA | NA |
| **BR 271** | F | >45 | Mest | NA | NA | Agreste | C | N | N | Y | Y | AA | CT | CC | GG | II |
| **BR 272** | M | >45 | White | 2-4 | 4 | Agreste | C | N | N | N | N | AA | CT | CC | GG | DI |
| **BR 273** | M | >45 | NA | 2-4 | >4 | Mata | B1 | N | Y | N | N | AA | CT | CT | AA | DI |
| **BR 274** | F | ≤45 | Mest | 1 | >4 | Sertão | C | N | Y | Y | Y | AA | CC | CC | GA | NA |
| **BR 275** | F | >45 | Mest | 1 | 4 | Mata | B1 | N | N | N | N | NA | TT | NA | NA | NA |

| | | | | | | | | | | | | | | | | | |
|---|---|---|---|---|---|---|---|---|---|---|---|---|---|---|---|---|---|
| **BR 276** | F | >45 | NA | 2-4 | 4 | Mata | C | N | Y | Y | Y | AC | TT | CC | AA | DI |
| **BR 277** | F | >45 | Mest | 1 | 4 | Mata | B1 | N | N | N | N | AA | TT | CT | GA | NA |
| **BR 278** | F | >45 | NA | >5 | >4 | Sertão | A | Y | N | N | N | AA | CC | NA | GA | DI |
| **BR 279** | F | >45 | Mest | 1 | 4 | Agreste | A | N | N | N | N | AA | TT | CC | AA | DD |
| **BR 280** | F | >45 | Black | 1 | >4 | RM | B1 | N | N | N | N | AC | CT | CC | AA | DI |
| **BR 281** | F | >45 | NA | 1 | 4 | Agreste | C | N | N | N | N | AA | CC | CC | GA | DD |
| **BR 282** | F | >45 | White | 2-4 | >4 | NA | B1 | N | N | N | N | AA | CT | TT | AA | DD |
| **BR 283** | M | >45 | Mest | 1 | 4 | Sertão | A | N | N | N | N | AC | TT | CT | GG | DD |
| **BR 284** | M | >45 | White | 2-4 | >4 | other | C | N | N | N | N | AA | CT | CC | AA | DI |
| **BR 285** | M | >45 | White | 2-4 | 4 | Mata | C | N | N | N | N | AA | TT | CT | GG | DI |
| **BR 286** | F | >45 | White | NA | 4 | Mata | A | N | N | N | N | NA | NA | NA | NA | DI |
| **BR 287** | F | ≤45 | NA | 1 | 4 | other | A | N | N | N | N | AA | TT | CT | GA | DI |
| **BR 288** | F | >45 | Mest | 1 | 4 | Sertão | B1 | N | N | N | N | AA | CT | CT | AA | DD |
| **BR 289** | F | >45 | Mest | 2-4 | 4 | other | C | N | N | N | N | AC | CT | CC | AA | II |
| **BR 290** | M | ≤45 | Mest | 1 | >4 | Sertão | B1 | N | N | N | N | AC | CT | TT | AA | DD |
| **BR 291** | M | >45 | White | >5 | 4 | other | B1 | N | N | N | N | AA | CC | CT | GA | DI |
| **BR 292** | F | >45 | Mest | 2-4 | >4 | Sertão | B1 | N | N | N | N | AA | CT | CC | GA | II |
| **BR 293** | F | ≤45 | Mest | 2-4 | >4 | Sertão | A | N | N | N | N | AA | CT | CT | GA | DI |
| **BR 294** | F | >45 | White | 1 | 4 | other | C | N | N | N | N | AA | CT | CC | GA | DI |
| **BR 295** | M | >45 | Mest | 1 | 4 | Agreste | C | N | N | N | N | AA | TT | CT | GG | DI |
| **BR 296** | M | ≤45 | Mest | 1 | 4 | Mata | B1 | N | N | N | N | AA | CC | CT | GG | II |
| **BR 297** | M | >45 | Mest | 2-4 | 4 | Mata | A | N | N | N | N | AA | TT | CT | GA | DI |
| **BR 298** | F | >45 | Mest | 1 | 4 | RM | A | N | N | N | N | AC | TT | TT | AA | DI |
| **BR 299** | F | >45 | Mest | 1 | 4 | Sertão | B1 | N | N | N | N | AA | TT | CC | GG | DD |
| **BR 300** | F | >45 | NA | 1 | 4 | Mata | B1 | N | N | N | N | AA | CT | CC | GA | II |
| **BR 301** | F | >45 | Black | 1 | 4 | Sertão | A | N | N | N | N | AA | CT | CT | AA | DD |
| **BR 302** | F | >45 | Mest | 2-4 | 4 | Mata | B1 | N | N | N | N | AC | CT | CT | AA | DD |
| **BR 303** | F | >45 | NA | NA | 4 | Agreste | B1 | N | N | N | N | AA | CT | CC | GG | II |
| **BR 304** | F | >45 | Mest | 1 | 4 | Mata | B1 | N | N | N | N | AA | CT | CC | GA | DI |
| **BR 305** | F | >45 | NA | 1 | 4 | Mata | B1 | N | N | N | N | AA | CC | CC | GA | II |
| **BR 306** | F | >45 | White | 1 | 4 | other | A | N | N | N | N | AC | TT | CT | GA | II |
| **BR 307** | F | >45 | Mest | 2-4 | >4 | other | B1 | N | N | N | N | AA | TT | CT | GA | DI |
| **BR 308** | M | >45 | White | >5 | 4 | other | C | N | N | N | N | AA | TT | CT | AA | DD |

| | | | | | | | | | | | | | | | | |
|---|---|---|---|---|---|---|---|---|---|---|---|---|---|---|---|---|
| **BR 309** | M | >45 | Mest | 2-4 | 4 | Mata | B1 | N | N | N | N | AC | CT | CC | GA | DI |
| **BR 310** | F | ≤45 | NA | >5 | >4 | Sertão | A | N | N | N | N | AA | CC | CT | GA | DD |
| **BR 311** | F | >45 | White | 1 | 4 | Agreste | C | N | N | N | N | AA | TT | CC | GA | DD |
| **BR 312** | F | >45 | White | 1 | 4 | Mata | A | N | N | N | N | AA | CC | CT | AA | DD |
| **BR 313** | F | >45 | Mest | 2-4 | >4 | Agreste | C | N | N | N | N | AC | CT | CC | GA | II |
| **BR 314** | M | >45 | Mest | 2-4 | 4 | Mata | B1 | N | N | N | N | AC | CT | CC | GA | DD |
| **BR 315** | M | >45 | Black | 2-4 | 4 | Mata | B1 | N | N | N | N | NA | CT | CC | GG | DD |
| **BR 316** | F | >45 | White | 1 | 4 | Mata | B1 | N | N | N | N | NA | TT | CC | GA | DI |
| **BR 317** | M | >45 | Mest | 2-4 | 4 | Mata | B1 | N | N | N | N | NA | CT | CT | GA | DD |
| **BR 318** | M | ≤45 | Mest | 1 | >4 | other | A | N | N | N | N | AC | CT | CT | AA | DD |
| **BR 319** | M | ≤45 | NA | NA | NA | Sertão | A | N | N | N | N | NA | TT | CT | GG | DI |
| **BR 320** | M | >45 | NA | 1 | 4 | Sertão | A | N | N | N | N | AA | CT | CC | AA | DD |
| **BR 321** | F | >45 | Mest | 1 | 4 | Mata | C | N | N | N | N | AC | CT | CC | GA | DD |
| **BR 322** | F | >45 | NA | NA | >4 | Agreste | B1 | N | N | N | N | AC | CT | CC | GA | DI |
| **BR 323** | F | >45 | White | 1 | 4 | RM | C | N | N | N | N | AC | CT | CC | AA | DI |
| **BR 324** | M | >45 | NA | NA | 4 | Mata | C | N | N | N | N | AA | TT | CC | GG | DI |
| **BR 325** | F | >45 | Mest | 1 | 4 | Mata | C | N | N | N | N | AA | CT | CC | GA | DI |
| **BR 326** | M | ≤45 | Mest | 1 | 4 | Mata | A | N | N | N | N | AC | TT | CC | GG | DD |
| **BR 327** | M | >45 | Mest | 1 | 4 | Agreste | B1 | N | N | N | N | AA | CT | CT | AA | DI |
| **BR 328** | F | >45 | NA | NA | 4 | Mata | B1 | N | N | N | N | AC | TT | CT | AA | DD |
| **BR 329** | M | >45 | Black | NA | NA | Agreste | B1 | N | N | N | N | AA | CT | CC | GA | DD |
| **BR 330** | M | ≤45 | NA | NA | NA | NA | A | N | N | N | N | AA | CT | CT | GA | DI |
| **BR 331** | F | >45 | Black | NA | NA | Mata | C | N | N | N | N | AC | TT | CC | GA | DD |
| **BR 332** | F | >45 | Black | 2-4 | 4 | Agreste | C | N | N | N | N | AA | TT | CC | AA | DI |
| **BR 333** | M | >45 | Mest | 1 | 4 | Sertão | A | N | N | N | N | NA | CT | CC | GA | II |
| **BR 334** | F | >45 | Mest | 1 | 4 | other | A | N | N | N | N | AC | TT | CC | AA | DI |
| **BR 335** | M | ≤45 | White | 1 | >4 | Sertão | A | Y | N | N | N | AA | CT | TT | AA | II |
| **BR 336** | F | >45 | Mest | 1 | 4 | Agreste | A | N | N | N | N | AC | TT | CT | GA | DI |
| **BR 337** | F | >45 | NA | NA | NA | NA | A | Y | N | N | N | AC | CT | CT | AA | DI |
| **BR 338** | F | >45 | Mest | 1 | 4 | other | C | N | Y | N | N | AC | TT | CT | AA | II |
| **BR 339** | F | >45 | NA | 1 | NA | RM | A | Y | N | N | N | AC | TT | CT | AA | II |
| **BR 340** | F | ≤45 | Mest | 1 | 4 | Mata | A | Y | N | N | N | AA | TT | CT | GG | DI |
| **BR 341** | F | >45 | White | 1 | 4 | Mata | C | N | Y | Y | N | AA | CT | CC | GA | DI |

| | | | | | | | | | | | | | | | | |
|---|---|---|---|---|---|---|---|---|---|---|---|---|---|---|---|---|
| **BR 342** | F | ≤45 | White | 2-4 | 4 | Sertão | A | Y | N | N | N | AA | CT | CC | GG | DD |
| **BR 343** | F | >45 | Mest | 1 | 4 | Mata | A | Y | Y | Y | N | AA | CT | CC | GG | DI |
| **BR 344** | F | >45 | NA | 1 | 4 | Mata | A | N | Y | N | N | AC | TT | CT | GA | DI |
| **BR 345** | M | >45 | NA | 1 | 4 | Agreste | A | N | N | N | N | AA | CT | CT | GG | II |
| **BR 346** | F | ≤45 | Mest | 1 | 4 | Sertão | A | N | N | N | N | AC | CT | CC | AA | DI |
| **BR 347** | F | >45 | Mest | <1 | 4 | Sertão | A | N | N | N | N | AA | TT | CT | GA | II |
| **BR 348** | M | >45 | Mest | 1 | 4 | Agreste | A | Y | N | N | N | AC | TT | CC | GA | II |
| **BR 349** | F | ≤45 | Mest | 1 | >4 | Sertão | A | Y | N | N | N | AC | TT | CC | GG | DD |
| **BR 350** | F | ≤45 | White | 1 | >4 | RM | A | Y | N | N | N | AA | CT | CC | GG | DD |
| **BR 351** | M | ≤45 | NA | 2-4 | NA | other | A | N | N | N | N | AA | CC | CC | GA | DD |
| **BR 352** | F | >45 | Mest | 1 | 4 | Sertão | A | N | N | N | N | AC | TT | CC | AA | DI |
| **BR 353** | F | ≤45 | Mest | >5 | >4 | Agreste | A | N | N | N | N | AC | TT | CC | AA | DI |
| **BR 354** | F | ≤45 | Mest | 1 | 4 | Mata | A | Y | N | N | N | AA | CT | CC | GG | DD |
| **BR 355** | M | >45 | White | >5 | >4 | other | A | N | N | N | N | AA | CC | CC | GA | DD |
| **BR 356** | M | >45 | White | 1 | 4 | Mata | A | Y | NA | NA | NA | AC | TT | CT | GA | DI |
| **BR 357** | F | >45 | Black | 1 | >4 | Agreste | A | Y | N | N | N | AC | TT | CT | GA | DI |
| **BR 358** | M | >45 | Mest | NA | 4 | Sertão | C | N | Y | Y | Y | AA | CT | CC | GG | DD |
| **BR 359** | F | >45 | Mest | 1 | 4 | Mata | C | N | Y | Y | N | AA | TT | CT | GG | DI |
| **BR 360** | F | >45 | Black | 1 | 4 | Mata | C | N | Y | Y | N | AA | TT | CT | GG | DI |
| **BR 361** | F | >45 | Mest | 1 | >4 | Mata | C | Y | Y | Y | N | AA | TT | CC | GA | DD |
| **BR 362** | F | ≤45 | NA | 1 | 4 | Mata | C | Y | Y | Y | Y | AC | CT | CC | AA | DI |
| **BR 363** | F | >45 | Mest | 1 | 4 | Mata | A | Y | Y | Y | N | AC | TT | CC | GA | DI |
| **BR 364** | M | >45 | White | 1 | 4 | Agreste | A | N | N | N | N | AA | CC | CC | AA | DI |
| **BR 365** | M | >45 | Mest | 1 | 4 | Mata | C | N | Y | Y | N | AC | CT | CT | GA | II |
| **BR 366** | M | ≤45 | White | 1 | 4 | other | C | N | Y | Y | Y | AA | CT | CT | GA | DI |
| **BR 367** | M | ≤45 | Mest | 2-4 | 4 | Mata | C | N | Y | Y | Y | AA | CT | CT | GA | DD |
| **BR 368** | F | >45 | Mest | 1 | 4 | other | C | Y | Y | N | Y | AA | TT | CC | GG | DI |
| **BR 369** | F | ≤45 | White | 2-4 | >4 | Sertão | A | Y | N | N | N | AA | CT | CC | AA | II |
| **BR 370** | F | >45 | Mest | 1 | 4 | Mata | A | N | N | N | N | AA | CC | CC | GG | DD |
| **BR 371** | M | ≤45 | White | <1 | 4 | Mata | A | N | N | N | N | AA | CT | CC | GG | II |
| **BR 372** | M | ≤45 | White | 1 | 4 | Mata | C | Y | NA | NA | NA | AC | TT | CC | GA | DI |
| **BR 373** | F | ≤45 | Mest | 1 | >4 | other | A | Y | N | N | N | AC | CT | CC | GA | DD |
| **BR 374** | F | ≤45 | Mest | <1 | 4 | Sertão | A | N | N | N | N | AC | TT | CC | GG | DI |

| | | | | | | | | | | | | | | | | |
|---|---|---|---|---|---|---|---|---|---|---|---|---|---|---|---|---|
| **BR 375** | F | NA | White | 2-4 | >4 | Sertão | B1 | NA | N | N | N | AA | TT | CC | GA | II |
| **BR 376** | F | >45 | White | <1 | >4 | Mata | C | N | Y | Y | N | AC | CT | CT | AA | DD |
| **BR 377** | M | >45 | Mest | 1 | 4 | Mata | A | N | N | N | N | AA | CT | CC | GG | DI |
| **BR 378** | F | >45 | NA | 2-4 | 4 | Sertão | A | Y | N | N | N | AA | CT | CT | GA | DI |
| **BR 379** | F | >45 | Mest | 1 | 4 | Agreste | A | N | Y | N | N | AC | TT | CC | GG | DI |
| **BR 380** | F | >45 | White | 2-4 | 4 | Sertão | A | Y | N | Y | N | AC | CT | CC | GA | II |
| **BR 381** | M | NA | Mest | 2-4 | 4 | RM | B1 | NA | N | N | N | AA | TT | CT | GG | DI |
| **BR 382** | F | >45 | Mest | NA | 4 | Agreste | A | N | N | N | N | AA | CT | CT | AA | DD |
| **BR 383** | F | >45 | Mest | 1 | 4 | Sertão | A | Y | N | Y | N | AA | CT | CC | GG | II |
| **BR 384** | F | >45 | White | 1 | 4 | Agreste | A | N | N | N | N | AA | CT | CT | GA | II |
| **BR 385** | M | ≤45 | Mest | 2-4 | 4 | Sertão | A | N | N | N | N | AA | NA | NA | GG | DI |
| **BR 386** | F | ≤45 | Mest | <1 | 4 | RM | A | N | N | N | N | AC | TT | CT | GA | II |
| **BR 387** | F | ≤45 | Mest | 1 | 4 | Agreste | A | Y | N | N | N | AA | TT | CT | GG | II |
| **BR 388** | M | ≤45 | Mest | 1 | 4 | Sertão | A | N | N | Y | N | AC | TT | CC | GA | DD |
| **BR 389** | F | >45 | White | 1 | 4 | Mata | A | N | N | N | N | AA | TT | NA | GA | II |
| **BR 390** | M | ≤45 | Mest | 1 | 4 | other | A | Y | N | N | N | AA | TT | CC | GG | DD |
| **BR 391** | F | ≤45 | Mest | 1 | 4 | other | A | N | N | N | N | AA | CT | CC | GA | DD |
| **BR 392** | F | ≤45 | Mest | 1 | >4 | Sertão | A | N | N | N | N | AA | CT | CT | AA | II |
| **BR 393** | F | >45 | Mest | 1 | 4 | Agreste | A | N | N | Y | N | AC | CT | CC | GA | DD |
| **BR 394** | F | ≤45 | Black | 1 | 4 | Agreste | A | Y | N | N | N | AC | TT | CT | AA | II |
| **BR 395** | F | ≤45 | Mest | <1 | 4 | Mata | C | N | Y | Y | Y | AA | TT | CC | GG | DD |
| **BR 396** | M | >45 | White | 1 | 4 | Mata | A | N | N | N | N | AA | CT | CT | GA | II |
| **BR 397** | F | >45 | White | 1 | NA | Mata | A | N | N | Y | N | AA | CC | NA | NA | DI |
| **BR 398** | F | >45 | Mest | 2-4 | 4 | other | A | N | N | Y | N | AA | CT | CC | GG | II |
| **BR 399** | M | ≤45 | White | 2-4 | 4 | Agreste | A | N | N | N | N | AC | CT | CC | GA | DI |
| **BR 400** | F | >45 | Black | 2-4 | 4 | Mata | A | N | N | N | N | AA | CT | CC | GA | DI |
| **BR 401** | F | >45 | Mest | 1 | 4 | RM | C | Y | N | Y | N | AA | CT | CT | AA | DI |
| **BR 402** | F | >45 | Mest | NA | 4 | RM | A | N | N | N | N | AA | CT | CC | GG | DI |
