# Supplementary material for: Influence of angiotensin II type 1 receptors and angiotensin-converting enzyme I/D gene polymorphisms on the progression of Chagas’ heart disease in a Brazilian cohort: Impact of therapy on clinical outcomes
Source: PLoS Negl Trop Dis. 2024 Nov 26;18(11):e0012703. doi: 10.1371/journal.pntd.0012703 (PMC11630595; doi:10.1371/journal.pntd.0012703)
Supplement: S3 Table — (DOCX) [file pntd.0012703.s003.docx]

**S2 Table.** Details of the TaqMan assays^a^ used for *AGTR1* SNP genotyping.

| **Gene** | **Location**^b^ | **SNP** | **A>a** | **dbSNP** | **TaqMan Assay ID** |
| --- | --- | --- | --- | --- | --- |
| ***AGTR1*** | chr3: 148741608 | +573 | C>T | rs5182 | C___8758668_10 |
| ***AGTR1*** | chr 3: 148697758 | -119 | C>T | rs275653 | C____950083_10 |
| ***AGTR1*** | chr3: 148706356 |  | A>G | rs2131127 | C__16268445_10 |
| ***AGTR1*** | chr3: 148742201 | +1166 | A>C | rs5186 | C___3187716_10 |

^a^ ThermoFisher Scientific, USA.

^b^ Location is based on GRCh38 Assembly build.

Abbreviations: A = ancestral allele, a = minor allele.
