# Supplementary figures and images for: Influence of angiotensin II type 1 receptors and angiotensin-converting enzyme I/D gene polymorphisms on the progression of Chagas’ heart disease in a Brazilian cohort: Impact of therapy on clinical outcomes
Source: PLoS Negl Trop Dis. 2024 Nov 26;18(11):e0012703. doi: 10.1371/journal.pntd.0012703 (PMC11630595; doi:10.1371/journal.pntd.0012703)

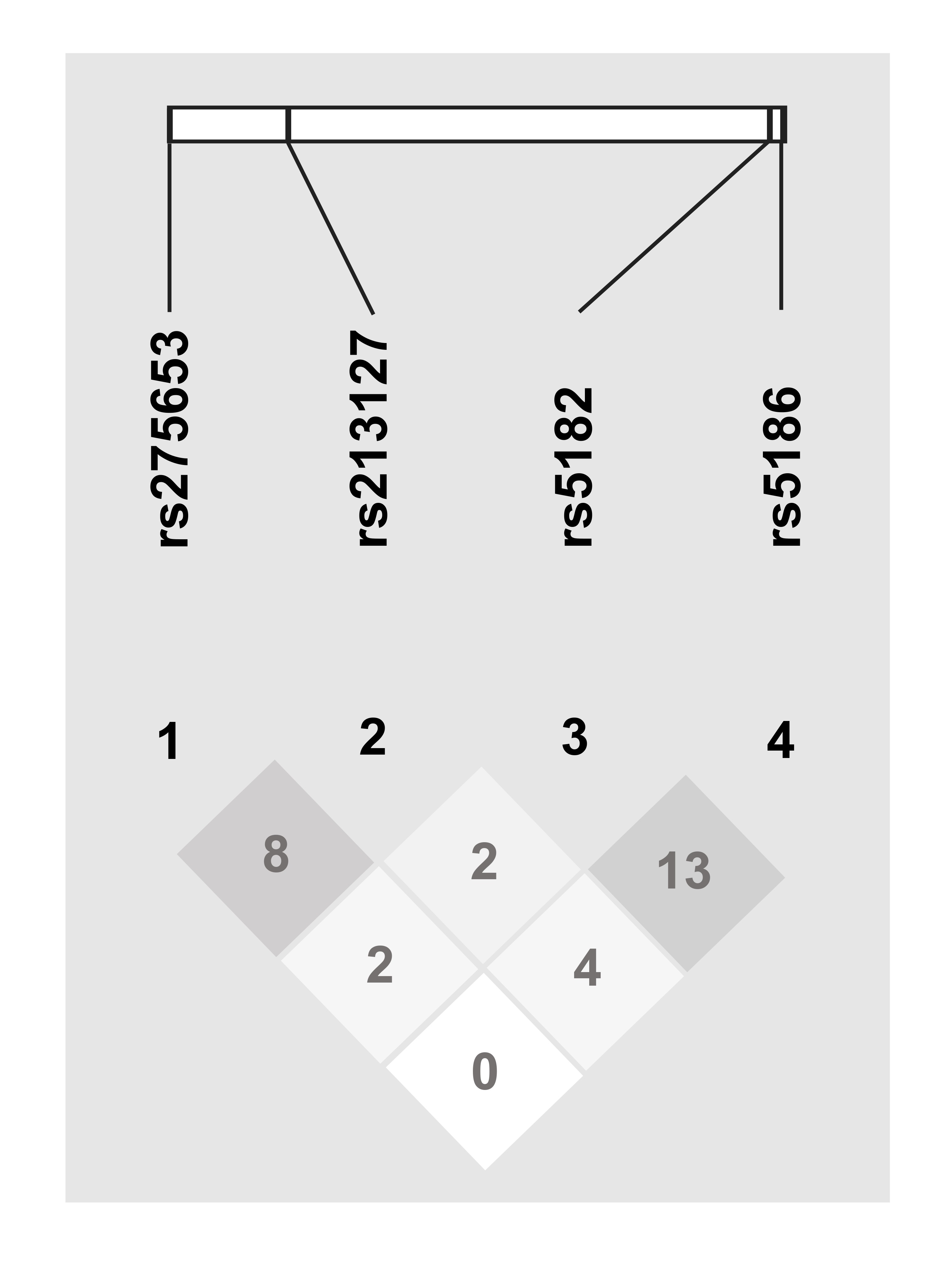

Supplement: S1 Fig — Values shown in each box and the intensity of shading are proportional to r2. (TIF) [file pntd.0012703.s004.tif]

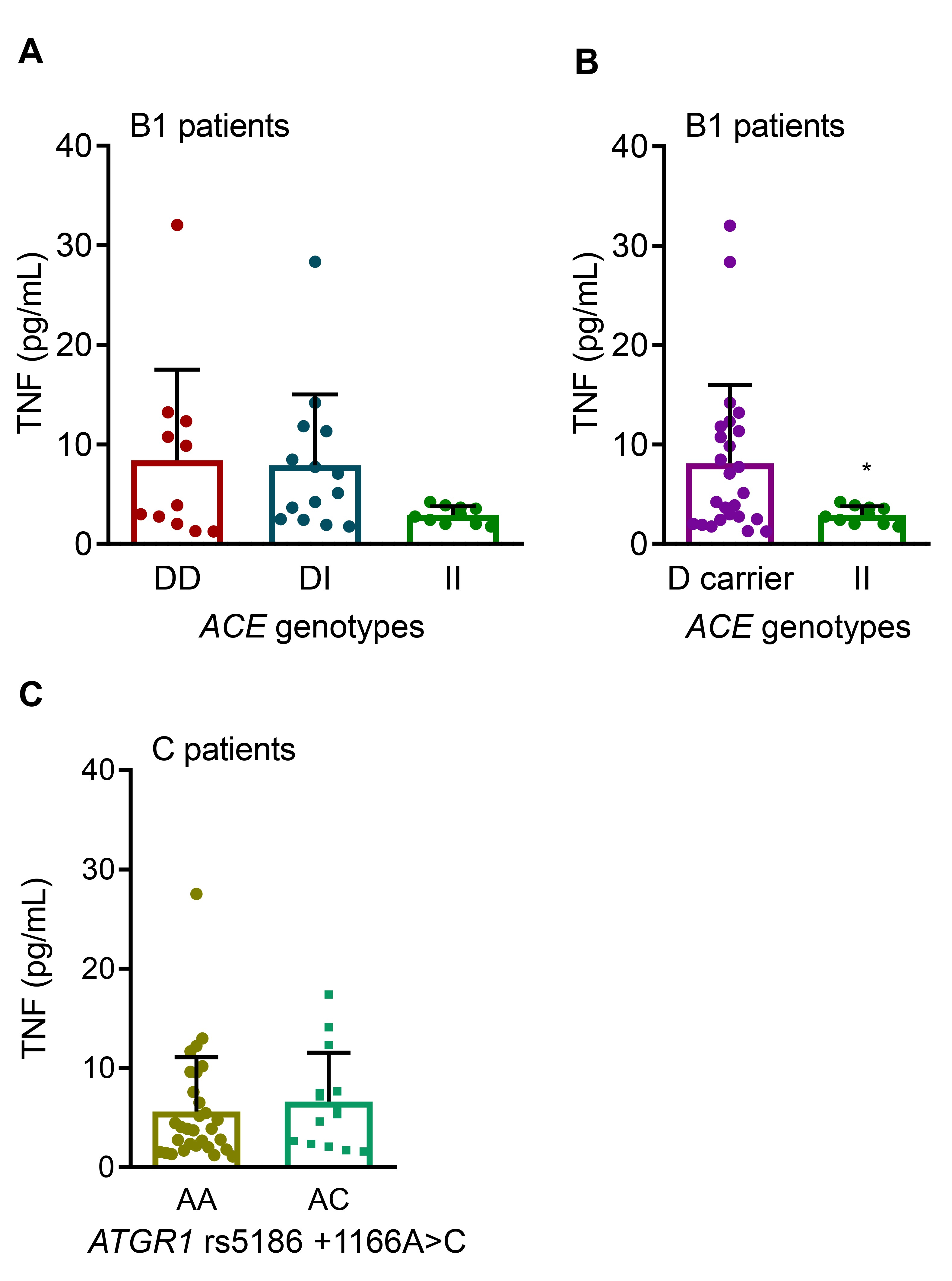

Supplement: S2 Fig — A. TNF serum concentrations (pg/mL) are shown in B1 group patients (mild Chagas’ heart disease) with ACE DD (n = 11), DI (n = 14) and II (n = 10) genotypes. B. TNF serum concentrations (pg/mL) in are shown B1 group patients classified as ACE D allele carriers compared to II genotype. C. TNF serum concentrations (pg/mL) in C group patients regarding the ATGR1 rs5186 +1166 AA (n = 30) and AC (n = 14) genotypes. Two-groups difference was analyzed with Student t-test. For data composed of more than two groups, the difference between groups was analyzed using the parametric one-way ANOVA test, corrected with Turkey post hoc test with multiple comparisons. Each dot represents a patient. Data show means ± SD. *, p < 0.05. (TIF) [file pntd.0012703.s005.tif]

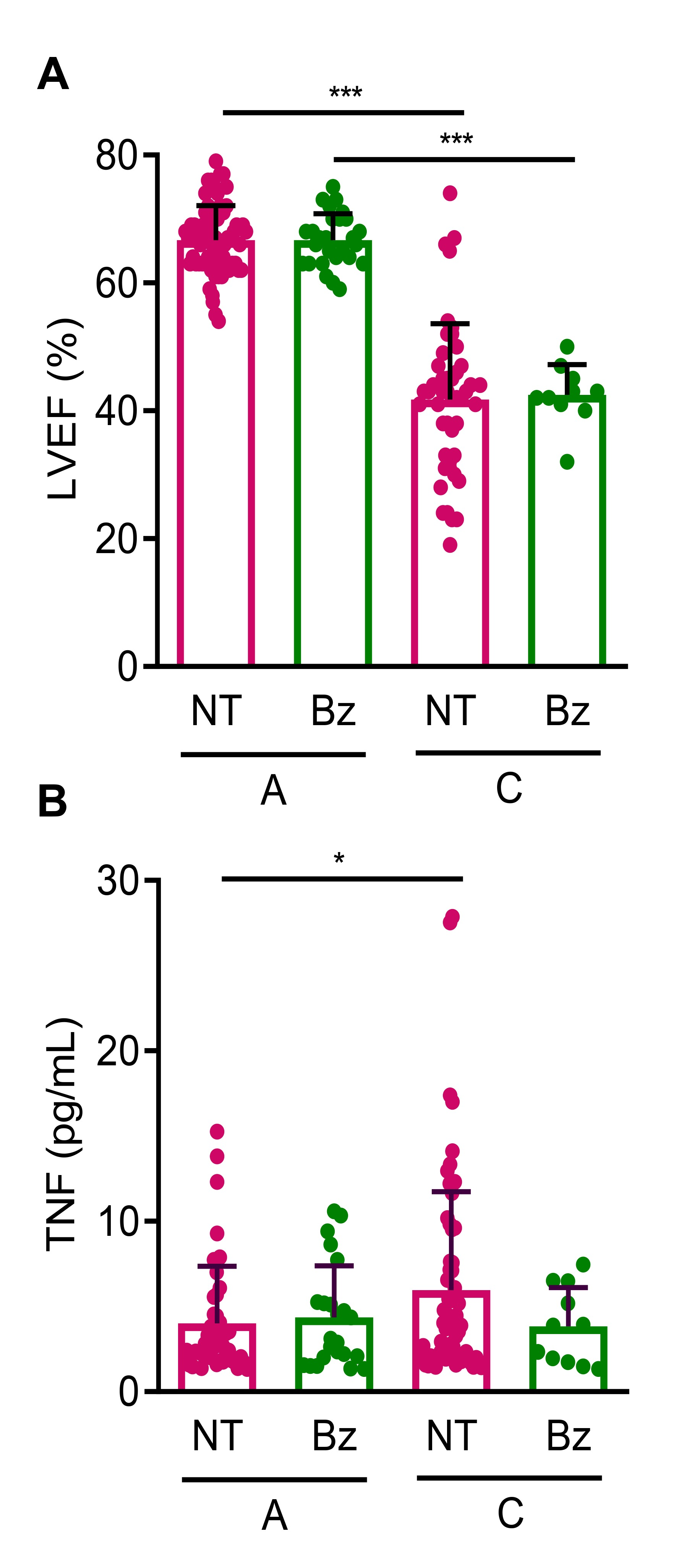

Supplement: S3 Fig — Cross-sectional observational study of groups A (non-cardiopathic) and C (severe Chagas’ heart disease) patients not-treated (NT) or administered with benznidazole (Bz) 1–20 years prior to inclusion in this study. A. TNF concentrations (pg/mL) in serum of A (n = 64, 27) and C (n = 47, 10) patients. B. Left ventricular ejection fraction (%; Simpson’s method) in A (n = 61, 23) and C (n = 43, 11) patients. For data composed of more than two groups, the difference between groups was analyzed using the parametric one-way ANOVA test, corrected with Turkey post hoc test with multiple comparisons. Each dot represents a patient. Data show means ± SD. *, p < 0.05, ***, p < 0.001. (TIF) [file pntd.0012703.s006.tif]
